# Ten new high-quality genome assemblies for diverse bioenergy sorghum genotypes

**DOI:** 10.3389/fpls.2022.1040909

**Published:** 2023-01-04

**Authors:** William G. Voelker, Krittika Krishnan, Kapeel Chougule, Louie C. Alexander, Zhenyuan Lu, Andrew Olson, Doreen Ware, Kittikun Songsomboon, Cristian Ponce, Zachary W. Brenton, J. Lucas Boatwright, Elizabeth A. Cooper

**Affiliations:** ^1^ Dept. of Bioinformatics & Genomics, University of North Carolina at Charlotte, Charlotte, NC, United States; ^2^ North Carolina Research Campus, Kannapolis, NC, United States; ^3^ Cold Spring Harbor Research Laboratory, Cold Spring Harbor, NY, United States; ^4^ United States Department of Agriculture - Agricultural Research Service in the North Atlantic Area (USDA-ARS NAA), Robert W. Holley Center for Agriculture and Health, Ithaca, NY, United States; ^5^ Carolina Seed Systems, Darlington, SC, United States; ^6^ Advanced Plant Technology, Clemson University, Clemson, SC, United States; ^7^ Dept. of Plant and Environmental Sciences, Clemson University, Clemson, SC, United States

**Keywords:** sorghum, genome assembly and annotations, pangenomics, bioenergy, structural variation

## Abstract

**Introduction:**

Sorghum (*Sorghum bicolor* (L.) Moench) is an agriculturally and economically important staple crop that has immense potential as a bioenergy feedstock due to its relatively high productivity on marginal lands. To capitalize on and further improve sorghum as a potential source of sustainable biofuel, it is essential to understand the genomic mechanisms underlying complex traits related to yield, composition, and environmental adaptations.

**Methods:**

Expanding on a recently developed mapping population, we generated *de novo* genome assemblies for 10 parental genotypes from this population and identified a comprehensive set of over 24 thousand large structural variants (SVs) and over 10.5 million single nucleotide polymorphisms (SNPs).

**Results:**

We show that SVs and nonsynonymous SNPs are enriched in different gene categories, emphasizing the need for long read sequencing in crop species to identify novel variation. Furthermore, we highlight SVs and SNPs occurring in genes and pathways with known associations to critical bioenergy-related phenotypes and characterize the landscape of genetic differences between sweet and cellulosic genotypes.

**Discussion:**

These resources can be integrated into both ongoing and future mapping and trait discovery for sorghum and its myriad uses including food, feed, bioenergy, and increasingly as a carbon dioxide removal mechanism.

## Introduction

Sorghum (*Sorghum bicolor* (L.) Moench) is a versatile, adaptable, and widely grown cereal crop that is valued for its efficiency, drought tolerance, and ability to grow in marginalized soils (Wayne Smith and Frederiksen, 2000). Present-day genotypes exhibit extensive genetic, phenotypic, morphological, and physiological diversity which stems both from their historical spread and modern breeding efforts aimed at optimizing sorghum for different end uses. With its wealth of naturally occurring genetic diversity and advantageous traits, sorghum has enormous value as a sustainable, fast-growing, and high-yielding bioenergy crop (Calviño and Messing, 2012).

Currently, sorghum is classified into four major ideotypes: grain, sweet, cellulosic, and forage. All of these types can be used in different bioenergy production methods (Wu et al., 2010), but to fully capitalize on their potential, it is essential to gain a better understanding of the genomic changes driving traits related to yield, carbon partitioning, and local adaptation. However, these types of traits are often difficult to dissect due to the nature of their underlying genetic architecture (Brachi et al., 2011), which can involve hundreds to thousands of genes and complex mutations that are not easily captured by short-read sequencing.

Structural genomic mutations are an important source of variation in many species, and can play key roles in phenotypic diversification and evolution. Advances in sequencing technology, especially the advent of high-throughput long-read sequencing, have made the detection of structural variants feasible in many plant species where these types of changes were previously uncharacterized. More recently, there has also been a surge in the generation of pan-genomic data for a number of important crop species, which has offered exciting new insights into the extensive diversity of these plants and the potential influence of complex structural mutations on agronomically important phenotypes (2022; Golicz et al., 2016; Zhang et al., 2019; Danilevicz et al., 2020; Zhou et al., 2020; Della Coletta et al., 2021; Hufford et al., 2021; Li et al., 2021).

Previous genomic work in sorghum has linked structural mutations to a number of key traits including dwarfing (Multani et al., 2003), juicy stalks (Zhang et al., 2018), chilling tolerance (Wu et al., 2019), and flowering time (Li et al., 2018). A whole-genome comparison of the sweet sorghum genotype ‘Rio’ with ‘BTx623,’ (a short-statured, early maturing grain sorghum) found hundreds of gene presence/absence variations (PAVs), several of which occurred among known sucrose transporters (Cooper et al., 2019). Furthermore, a genome-wide association study (GWAS) exploring the genetic architecture of bioenergy-related traits found that a large deletion in a sorghum-specific iron transporter was linked to stalk sugar accumulation (2020; Brenton et al., 2016). Most recently, we undertook a broad survey of genome-wide deletions in a panel of nearly 350 diverse sorghum accessions, and found large deletions in multiple genes related to biotic and abiotic stress responses that were unique to particular geographic origins, and appeared to play a role in local adaptation (Songsomboon et al., 2021).

Taken together, these results suggest that unraveling complex traits in sorghum and other crops will require a comprehensive picture of both structural and single nucleotide mutations. In this study, we have expanded on the recently published Carbon-Partitioning Nested Association Mapping (CP-NAM) population that was developed and publicly released as a key genetic resource for the characterization and improvement of sorghum for multiple different end uses (2022; Boatwright et al., 2021; Kumar et al., 2022). We generated high-quality *de novo* genome assemblies for 10 of the CP-NAM parents and used these genomes to identify millions of novel variants, including a number of large structural variants (SVs) occurring in genes or pathways that could be essential for optimizing sorghum as a bioenergy feedstock.

## Materials and methods

### Sample collection and sequencing

Seeds for each genotype were ordered from the U.S. Department of Agriculture’s Germplasm Resource Information Network (GRIN)(https://www.ars-grin.gov/) and grown in the greenhouses at the North Carolina Research Campus (NCRC) in Kannapolis, NC. High-molecular-weight DNA was extracted from each sample using a modified high-salt CTAB extraction protocol (Inglis et al., 2018). Purified DNA was sent to the David H. Murdock Research Institute (DHRMI) for quality control, library preparation, and sequencing on a PacBio Sequel I system.

### *De novo* assembly

Raw subreads for each genotype were combined and converted to FASTQ format using the bam2fastx toolkit from PacBio. Reads were then corrected, trimmed, and assembled using Canu(v2.1.1) (Koren et al., 2017). For one of the genotypes, ‘Grassl’, Canu failed to produce contigs due to reduced read coverage after trimming, so the final assembly was instead produced using Flye(v2.9) with the Canu corrected reads (Kolmogorov et al., 2019).

The resulting contigs for all genotypes were scaffolded into chromosomes using RagTag (v2.1.0) (Alonge et al., 2021) and the parameters ‘-r -g 1 -m 10000000’. Contigs were ordered based on their alignment to the BTx623 v3.1 reference genome (Paterson et al., 2009) with minimap2 (Li, 2018). RagTag was run *without* the correction step to avoid unnecessary fragmentation of the contigs and unplaced contigs were discarded. Assembled genome metrics were assessed both before and after scaffolding using QUAST(5.2.0) (Gurevich et al., 2013).

### Annotation

Protein and non-coding genes were annotated by building a pan-gene working set using representative pan-gene models selected from a comparative analysis of gene family trees from 18 Sorghum genomes (McCormick et al., 2018; Deschamps et al., 2018; Cooper et al., 2019; Wang et al., 2021; Tao et al., 2021) sourced from SorghumBase(https://www.sorghumbase.org/). This pan-gene representative was propagated onto the 10 sorghum genome assemblies using Liftoff (v1.6.3) (Shumate and Salzberg, 2021) with parameters(-a 0.95 -s 0.95 -p 20 -copies -cds -polish). The gene structures were updated with available transcriptome evidence from Btx623 using PASA (v2.4.1) (Haas et al., 2003). Additional improvements to structural annotations were done in PASA using full length sequenced cDNAs and sorghum ESTs downloaded from NCBI using the query (EST[Keyword]) AND sorghum[Organism]. The working set was assigned Annotation Edit Distance(AED) scores using MAKER-P (v3.0) (Campbell et al., 2014) and transcripts with AED score < 1 were classified as protein coding. Those with AED=1 were further filtered to keep any non-BTx623 based models with a minimum protein length of 50 amino acids and a complete CDS as protein coding. The remaining models with AED=1 were classified as non-coding. Gene ID assignment was made as per the existing nomenclature schema established for Sorghum reference genomes (McCormick et al., 2018).

On average, approximately 55 thousand working sets of models were generated for each sorghum line, out of which an average of 41 thousand were coding and roughly 13 thousand were non-coding (**Supplementary Table 1**). More than half (61%) of the protein coding models mapped to a BTx623 reference gene, along with 23% of the non-coding models (**Supplementary Figure 1A**). On average ~42% single exon genes come from the reference BTx623 genome, while ~52% come from non-BTX623 lines. ~92% of the single exon genes that are not found in non-sorghum reference genomes, are found in two or more sorghum accessions. ~29% of these have a supporting AED score of less than 1 (**Supplementary Figure 1B**). Functional domain identification was completed with InterProScan (v5.38-76.0) (Jones et al., 2014). TRaCE (Olson and Ware, 2020) was used to assign canonical transcripts based on domain coverage, protein length, and similarity to transcripts assembled by Stringtie. Finally, the protein coding annotations were imported to Ensembl core databases, verified, and validated for translation using the Ensembl API (Stabenau et al., 2004).

In order to assign gene ages, protein sequences were aligned to the canonical translations of gene models from *Zea mays*, *Oryza sativa*, *Brachypodium distachyon*, and *Arabidopsis thaliana* obtained from Gramene release 62 (Tello-Ruiz et al., 2020) using USEARCH v11.0.667_i86linux32 (Edgar, 2010). If there was a hit with minimum sequence identity of 50% (-id 0.5) to an *Arabidopsis* protein, the gene was classified as being from Viridiplanteae, if there was a hit to rice the gene was classified as Poaceae, and if a hit was to maize the gene was classified as Andropogoneae. If there were no hits then the gene was classified as sorghum specific.

### Repeat analysis

Transposable elements (TEs) were identified and annotated in each genome using EDTA (Ou et al., 2019). TE-greedy-nester (Lexa et al., 2020) was used to further annotate both complete and fragmented Long Terminal Repeat (LTR) retrotransposons. Sequence divergence in the LTR regions was used to estimate retrotransposon age (SanMiguel et al., 1998; Jedlicka et al., 2020). The left and right LTR sequences were extracted from the assembled genomes using the coordinates reported by TE-greedy-nester and the getfasta tool from the BEDTools package(v2.29.0) (Quinlan and Hall, 2010). For each TE, the two LTR sequences were aligned using Clustal-W (Thompson et al., 1994) as implemented in the R package msa (Bodenhofer et al., 2015). Genetic distance was calculated based on the K80 model using the dist.dna function in the R package phangorn (Schliep, 2011). The time of divergence was calculated based on the equation T=K/(2 * r) (Bowen and McDonald, 2001), where T is the time of divergence, K is the genetic distance, and r is the substitution rate. A value of 0.013 mutations per million years was used for r, consistent with the molecular clock rate for LTRs estimated in rice (Ma and Bennetzen, 2004). To determine if any of the shell genes across all the genotypes had overlaps with TEs, a custom python script was used to match the annotated shell gene coordinates with TE coordinates identified by TE-greedy-nester (Lexa et al., 2020). A flanking sequence of 1000bp upstream and downstream was considered. In order to find the overlaps, only the contigs that were placed into chromosomes by RagTag(v2.1.0) (Alonge et al., 2021) were included since the unplaced contig sequences were not a part of TE-greedy-nester analysis.

### Variant calling

Filtered and scaffolded reads were realigned to the BTx623 reference genome using the nucmer program from the MUMmer(v4.0) package (Delcher et al., 2003; Marçais et al., 2018) with the following parameters ‘-c 100 -b 500 -l 50’. Alignments were filtered using the delta-filter program from the MUMmer package with the parameters ‘-m -i 90 -l 100’ and converted to coordinate files using show-coords with the parameters ‘-THrd’. Variants were then called using Syri(v1.6) (Goel et al., 2019).

Individual Syri VCF files were split by variant type (SNPs, Deletions, Insertions, Inversions, and Translocations) resulting in separate files for each variant type for each genotype. Insertions or deletions smaller than 50 bp were classified as small indels while those equal to or larger than 50 bp were classified as SVs. More complex SV types that could not be validated with raw reads were not considered for further analysis.

The Syri program produces a nonstandard VCF format which includes information on variants from overlapping syntenic blocks. This can result in duplicated variants and fragmented insertions that must be addressed before subsequent analysis with downstream tools. Duplicates of existing variants were removed for all variant types, and fragmented insertions were combined into single variants (**Supplementary Figure 2**). These processed variant files were then zipped and indexed using bgzip and tabix (Li et al., 2009) and then merged across genotypes using the merge function from the bcftools package with the parameters ‘-0 -I ‘ChrB:join,Parent:join,DupType:join,modified:join’ -O v’. This resulted in one variant file for each type of variant that included the genotypes for all individuals. Insertions, deletions, and SNPs were then annotated using SIFT (v2.4) (Vaser et al., 2016) and the BTx623 version 3.1.1 annotation to identify overlap with genes for insertions and deletions and missense prediction for single nucleotide variants.

### Phylogeny

Gene PAVs was called from pan-gene lift-off annotation information using custom python scripts. As per default liftoff parameters, gene presence was identified with a threshold of 95 percent similarity. PAVs for each genotype were encoded as a binary vector (with 0 indicating gene absence, and 1 indicating presence). Distance between genotypes was then calculated using the dist() function from the stats(v3.6.2) package in R using the Jaccard distance, and a phylogenetic tree was constructed using the NJ() function from the phangorn package. The SNP phylogeny used to confirm the PAV phylogeny was created using SNPs called from the program Syri. Similar to the PAV tree, this phylogeny was built based on a presence/absence binary matrix of SNPs. Genetic distance was calculated using the dist() function and the NJ() function in R.

### Gene ontology analysis

Gene ontology (GO) terms for genes affected by large insertions and deletions or nonsynonymous SNPs were curated from the publicly available annotation information file associated with BTx623 v3.1.1 in phytozome (https://phytozome-next.jgi.doe.gov/). GO enrichment analysis was performed using the R package topGO(v1.0) (Alexa and Rahnenfuhrer, 2016). The classic Fisher’s Test was used to assess significance of enriched terms, and terms with a p-value <0.05 were considered significant and kept for further analysis. Redundant and highly similar GO terms were defined and reduced based on semantic similarity using the R packages AnnotationForge (Carlson and Pages, 2022) and rrvgo (Sayols, 2020).

## Results

### Assembly quality and characteristics

**Figure 1:**
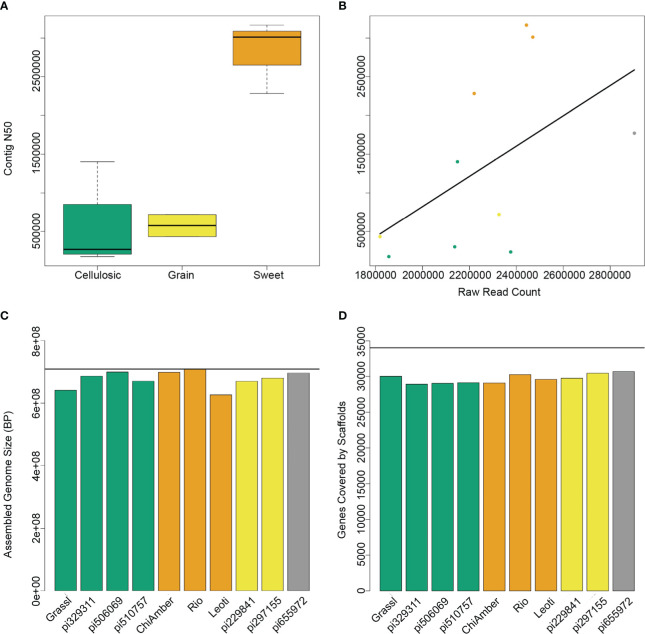
Assembly metrics for 10 sorghum genotypes. **(A)** Contig N50 levels for different ideotypes show higher contiguity for sweet genotypes. **(B)** Raw read counts prior to assembly are highly correlated with contig N50, and sweet genotypes (orange) have higher read counts than cellulosic (green) or grain (yellow) genotypes. **(C)** Assembled genome size after scaffolding and filtering for each genotype shows that despite differences in mean contig size, the final assemblies for both sweet and non-sweet types are very close to the expected reference genome size (horizontal black line). **(D)** The number of BTx623 genes contained within the final scaffolds is very similar across all genotypes regardless of type.

To capture the genetic diversity of bioenergy sorghum, we sequenced the parents of the previously established CP-NAM population, which included globally diverse genotypes representative of sweet, cellulosic, grain and forage type bioenergy sorghums (Boatwright et al., 2021) (**Table 1**). The initial contig-level assemblies showed a range of N50 values, with the lowest being 176 kb and the highest at over 3 Mbp (**Supplementary Table 2**). The three sweet genotypes in particular had a higher number of raw reads and more contiguous assemblies than the other types (**Figures 1A, B**), most likely as a result of differences in the effectiveness of the extraction protocol. After scaffolding and filtering unplaced contigs, all 10 genotypes showed similar levels of high contiguity, with final assembly sizes that were 90-98% the size of the BTx623 reference genome and over 90% of known BTx623 genes contained within the scaffolds (**Figures 1C, D**).

**Table 1:** Genotype origins, races, and types.

Name	Alternate ID	Race	Origin	Type
Grassl	PI 154844	Caudatum	Uganda	Sweet & Cellulosic
PI 329311	IS 11069	Durra	Ethiopia	Cellulosic
PI 506069	Mbonou	Guinea-bicolor	Togo	Cellulosic
PI 510757	AP79-714	Durra	Cameroon	Cellulosic
Chinese Amber	PI 22913	Bicolor	China	Sweet
Rio	PI 563295	Durra-caudatum	USA	Sweet
Leoti	PI 586454	Kafir-bicolor	Hungary	Sweet
PI 229841	IS 2382	Kafir	South Africa	Grain
PI 297155	IS 13633	Kafir	Uganda	Grain, Forage
PI 655972	Pink Kafir	Kafir	USA	Forage

Information adapted from GRIN and (Boatwright et al., 2021).

### Gene annotation

**Figure 2:**
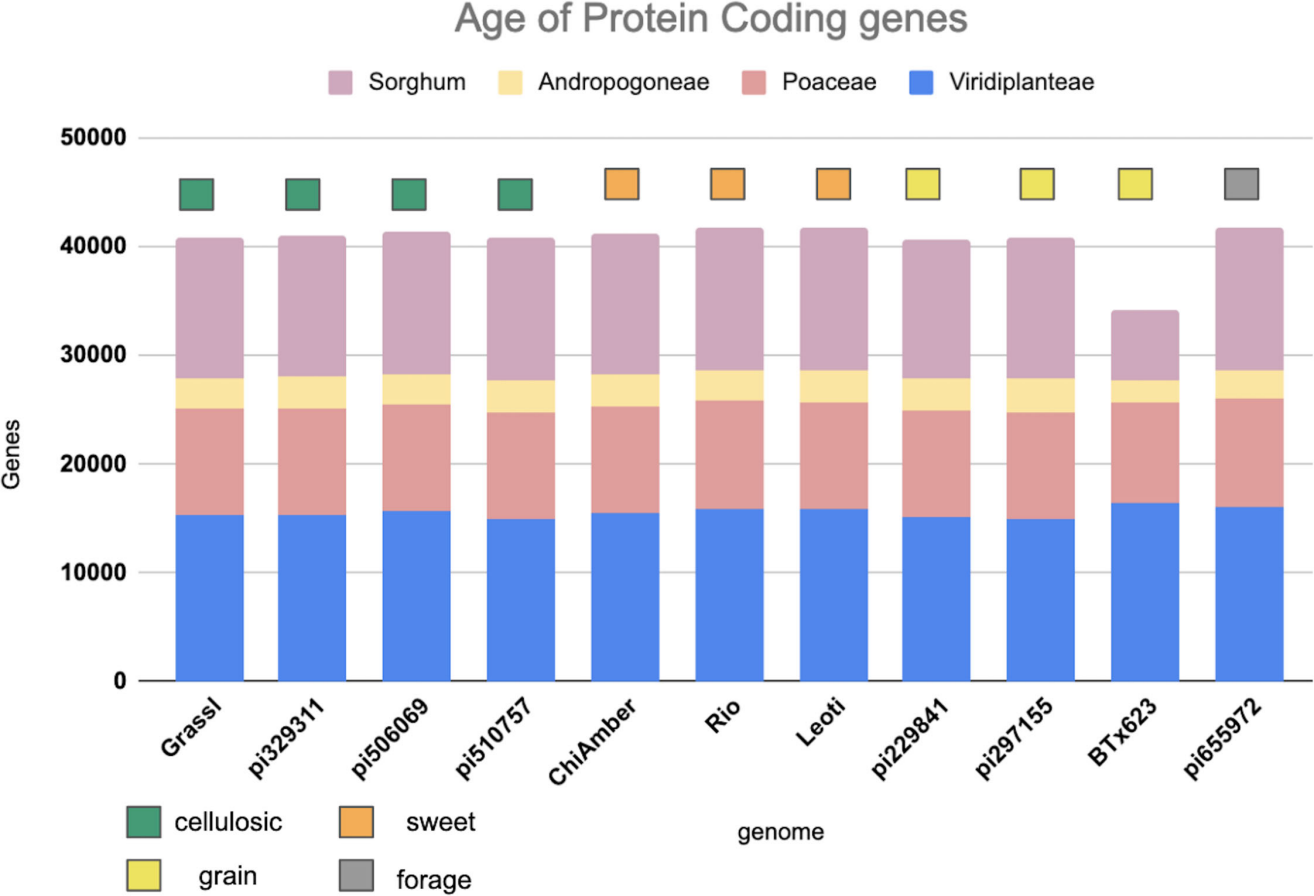
Age of protein coding genes among the sorghum lines based on minimum sequence identity. Bar color indicates the level of phylogenetic conservation, with blue indicating genes conserved across monocots and dicots; peach indicating the proportion of genes shared among the grasses; yellow indicating the proportion of genes shared between sorghum and maize, and light purple representing the proportion of sorghum-specific genes.

Genes shared across deeper evolutionary time scales were more conserved than sorghum-specific genes (**Figure 2**). The sweet genotypes show slightly more conserved genes when compared to other genotypes (**Figure 2**). Out of 62,044 genes annotated in the pan-genome, around 36.69 percent(22,762 genes) were found to be core to all genotypes, 50.32 percent(31,218 genes) were shell genes (present in more than one genome, but not all of the genomes), and 12.99 percent(8,064 genes) were found to be cloud genes (unique to a single genome) (**Supplementary Figure 3A**). The majority of shell genes were present in 9 of 10 genomes, with the second largest proportion of shell genes being present in 2 of 10 genomes (**Supplementary Figure 3B**). Of shell genes identified, 44 and 45 were identified to be exclusive to all sweet and all non-sweet genotypes respectively. Only 1-2 percent of shell genes in each genotype overlapped with or were flanked by LTRs, indicating that transposable element activity was not mediating the majority of observed gene content variation (**Supplementary Table 3**).

### Genomic landscape of variation

**Figure 3:**
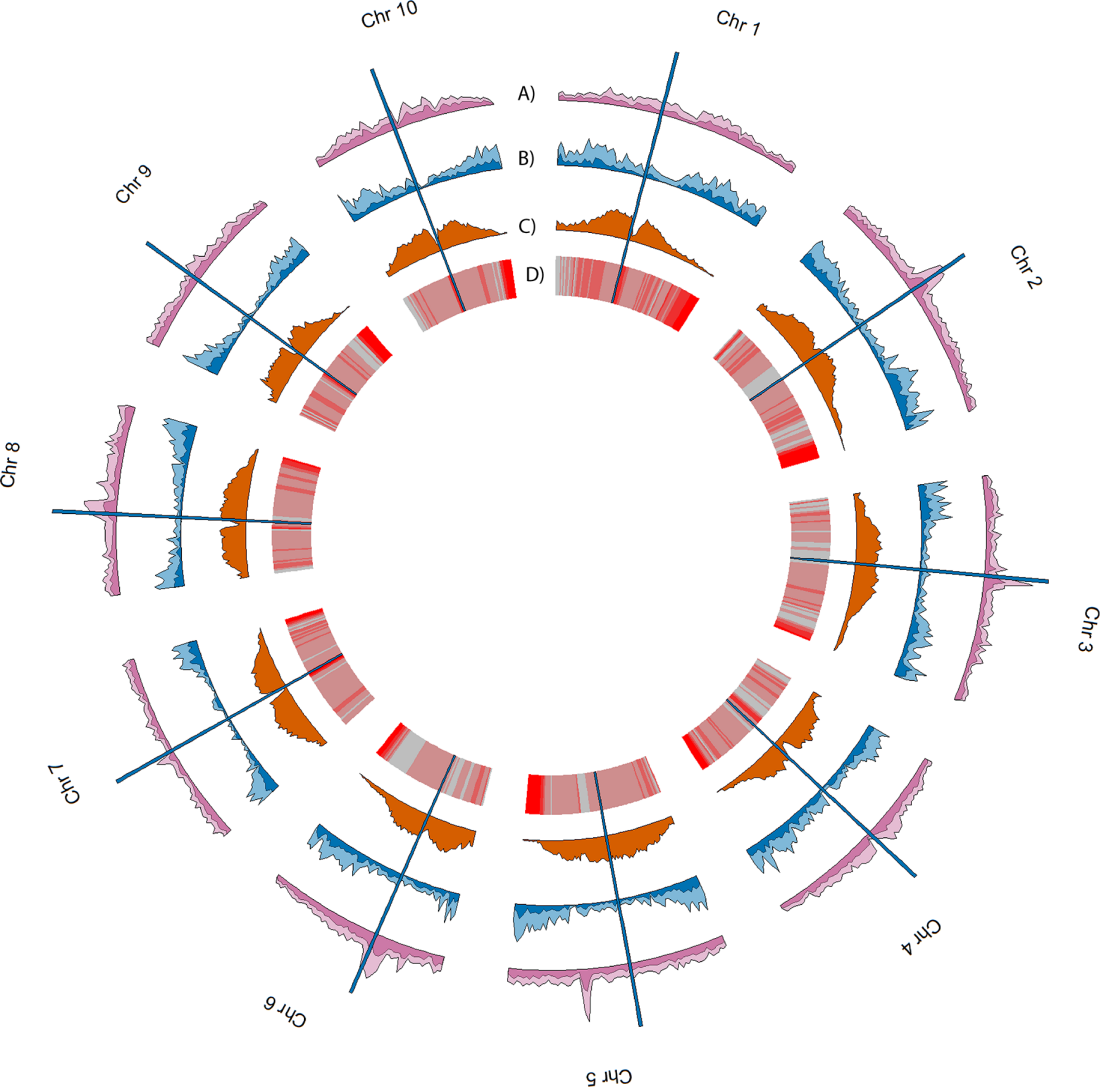
Genomic landscape of variation averaged across the 10 genomes. Density estimates in tracks A-C were performed in 1Mb non-overlapping sliding windows. **(A)** and **(B)** respectively show average SNP density and average SV density, with lighter colors indicating cloud variants and darker colors indicating shell and core variants. **(C)** shows the average TE density, and **(D)** shows TE age averaged across 1Mb sliding windows. Red indicates younger TEs while gray indicates older. Vertical blue bars spanning all tracks indicate the approximate position of the centromeres of each chromosome.

Over 10.5 million single nucleotide variants were called across the 10 genomes, as well as over 7.4 million small indels and over 24 thousand large structural variants (insertions and deletions ≥ 50 bp) (**Figure 3**, **Tables 2**, **3**). Well over half (~65%) of these variants were defined as cloud variation (**Table 3**), while the remaining variants were mostly shell. Only a small handful of core variants were present in all of the genotypes except the BTx623 reference. Phylogenetic relationships were inferred using gene presence/absence to estimate genetic distance (**Supplementary Figure 4A**), demonstrating that sweet, cellulosic, and grain genotypes come from separate clades within the category of bioenergy-type sorghum. These results were confirmed by SNP phylogeny (**Supplementary Figure 4B**).

**Table 2:** Variants found in each NAM parent genotype.

Genotype	Deletions (bp>=50)	Insertions (bp>=50)	Indels (bp<50)	SNPs	Nonsynonymous
Grassl	2,721	1,714	976,703	2,659,850	37,265
PI 329311	3,560	1,956	1,319,281	3,321,035	47,482
PI 506069	3,531	1,865	888,425	3,003,469	47,555
PI 510757	2,952	1,919	1,593,228	2,859,852	44,168
Chinese Amber	3,560	1,744	994,023	2,975,137	48,780
Rio	2,563	1,791	717,304	2,119,637	35,714
Leoti	3,279	1,435	785,360	2,790,452	43,473
PI 229841	2,830	1,490	1,447,030	2,546,090	41,679
PI 297155	2,412	1,335	1,151,594	2,052,203	34,863
PI 655972	2,401	1,113	631,705	1,953,106	32,758

**Table 3:** Core vs. Shell vs. Cloud variants.

Type	Deletions	Insertions	Total SVs	Indels	SNPs
**Core**	34	28	62	12,231	103,065
**Shell**	6,306	2,250	8,556	1,246,552	5,245,181
**Cloud**	7,855	8,232	16,087	6,195,713	5,416,344
**Total**	**14,195**	**10,510**	**24,705**	**7,454,496**	**10,764,590**

### Genes affected by structural variants and SNPs

**Figure 4:**
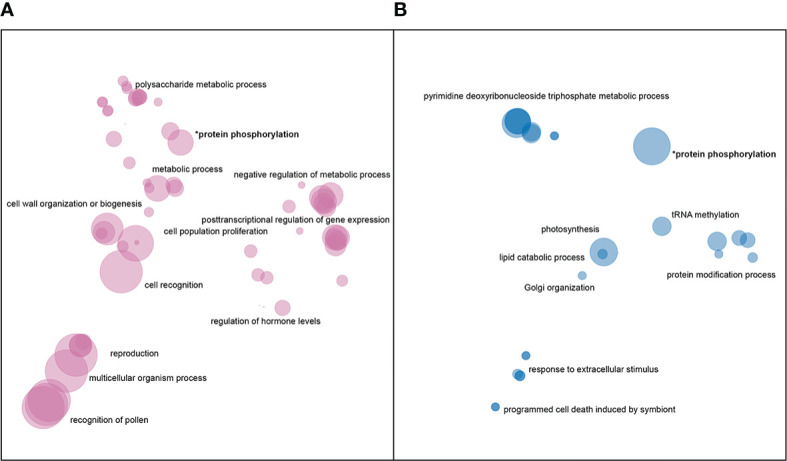
Enriched GO terms for genes impacted by **(A)** nonsynonymous SNPs and **(B)** large SVs. GO terms in each dataset were clustered and plotted based on semantic similarity as described in the Materials and Methods. Circle size is proportional to p-value, with larger circles indicating more significant terms.

There was a total of 171,000 SNPs that were found to be both located in genic regions and encoding nonsynonymous variants, and more than 2.5 thousand large SVs present in genic regions. GO enrichment analyses of affected genes revealed that SNPs and SVs tended to impact distinct categories of genes (**Figure 4**), with protein phosphorylation being the only significant category to appear in both datasets.

In addition to protein phosphorylation, genes impacted by large insertions or deletions showed enrichment in GO categories related to Golgi vesicle transport, photosynthesis, nucleoside metabolism, protein modifications, and programmed cell death (**Figure 4B**). Nonsynonymous SNPs, on the other hand, were enriched in genes involved in pollen-pistil interactions, cell wall biogenesis, cell proliferation, posttranscriptional regulation and polysaccharide metabolism (**Figure 4A**).

### Repeat analysis

**Figure 5:**
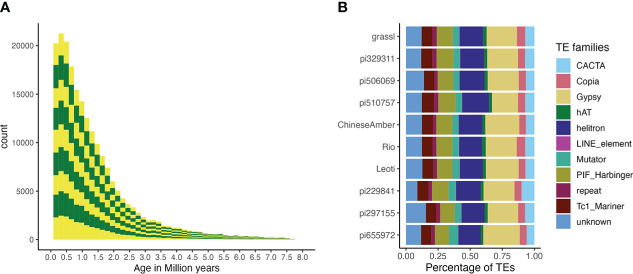
TE age and composition. **(A)** A stacked bar plot describing the distribution of TE counts by age across all genotypes. Alternating colors indicate different genotypes, and distributions are stacked in the order of the labels in figure 5b (i.e., the bottom yellow distribution shows the TE age frequencies for pi655972, while the top shows the distribution for Grassl)., T he Y-axis is number of TEs and the X-axis is their age in millions of years. **(B)** The proportion of superfamilies of TEs based on average counts of each superfamily across all genomes.

Overall, the TE composition was highly similar across all 10 genotypes (**Figures 5**, **3**), with the LTR-Gypsy superfamily comprising the majority of elements. The age analysis revealed an abundance of younger TEs, with a mean age of 1.28 million years old along with a high frequency of very young TEs approximately 0.1 million years old and very few old TEs (6-8 million years) (**Figure 5**; **Supplementary Figure 5**). Most (97.5%) of the TEs were non-nested, with TE-greedy-nester reporting the presence of only a handful (2.5%) of nested TEs. The overall distribution of TE age followed a similar pattern across all of the genotypes, with younger TEs being randomly distributed throughout the genome (**Figure 3**, **Supplementary Figure 6A-J**) as previously observed by (Paterson et al., 2009).

### Differences in sweet and non-sweet genotypes

**Figure 6:**
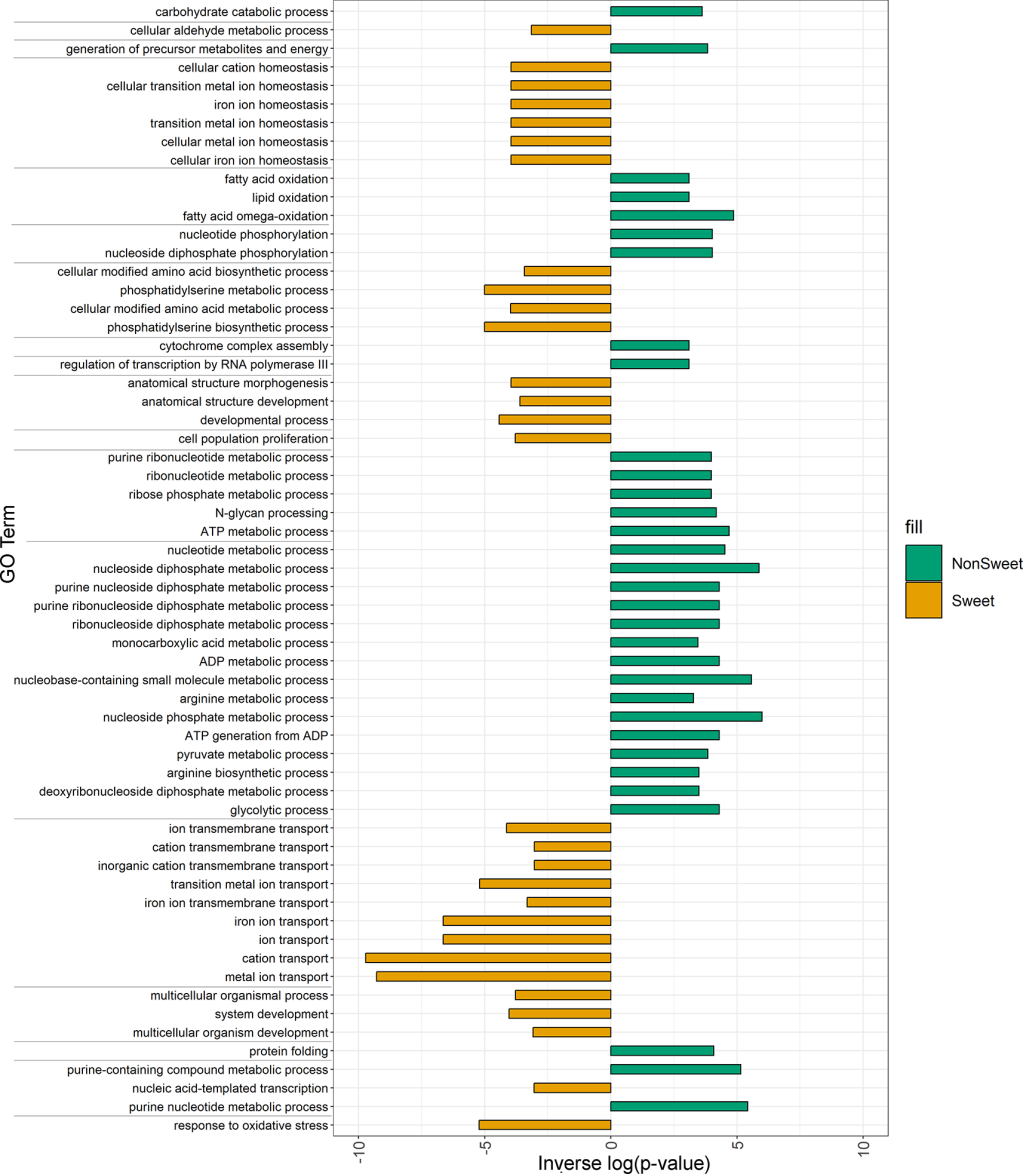
Enriched GO terms for genes impacted by SVs and Indels in both Non-Sweet and Sweet Genotypes. Orange bars indicated gene categories in sweet genotypes that were significantly impacted (p<0.05). Green bars indicated gene categories in non-Sweet genotypes that were significantly impacted (p<0.05). The length of each bar corresponds to significance (-log(p-value)). Terms have been clustered and sorted based on semantic similarity.

Structural variants that were present in all three sweet genotypes (Leoti, ChineseAmber, and Rio) but either absent from or rare among non-sweet genotypes, were significantly enriched among genes with functions related to metal ion transport, in particular iron ion transport, as well as genes involved in oxidative stress response, cell cycle arrest, and phosphatidylserine biosynthetic processes. Conversely, variants found only in all of the non-sweet genotypes tended to impact very different categories of genes, such as those involved in glycolytic processes, cytochrome assembly, and both RNA and DNA regulation (**Figure 6**).

## Discussion

Unraveling the molecular mechanisms controlling complex traits such as carbon partitioning, yield, and stress response is an essential step for crop improvement efforts aimed at creating effective and sustainable bioenergy feedstocks for the future. However, not only do these types of traits often involve changes in large numbers of genes, but an ever-increasing number of pan-genomics studies in crop plants have demonstrated that these changes can encompass complex structural mutations in addition to SNPs (2022; Cooper et al., 2019; Zhang et al., 2019; Brenton et al., 2020; Zhou et al., 2020; Hufford et al., 2021; Songsomboon et al., 2021). Therefore, the development of multiple reference-quality genomes within crop species is critical to the exploration of complex genetic architectures and has clear benefits when compared to a single reference genome, especially in the case of larger structural variants (Della Coletta et al., 2021). By *de novo* assembling 10 new high-quality genomes for the parents of the CP-NAM population (Boatwright et al., 2022), we have been able to uncover millions of novel variants, including thousands of large insertions and deletions.

Importantly, we found that SVs within coding regions impacted different types of genes compared to SNPs, highlighting the importance of incorporating both into future trait mapping studies. Many nonsynonymous SNPs that were segregating among the genotypes occurred in gene categories that have previously been linked to carbon allocation in sorghum and other closely related species. For instance, protein phosphorylation induces key signaling cascades in plants that control a variety of processes, and protein kinases have been shown to be highly differentially expressed in both sweet sorghum (Cooper et al., 2019) and sugarcane (Waclawovsky et al., 2010) during stem sugar accumulation. Similarly, genes involved in the regulation of plant hormones such as auxin were also enriched for non-coding SNPs, and these pathways are known to be essential for vegetative plant growth and stem elongation, both of which are key phenotypes for biomass accumulation (Kebrom et al., 2017).

Like SNPs, gene-impacting SVs were also found to affect many genes related to protein phosphorylation; in fact, this was the top category among genes containing large variants. But other categories enriched for high-impact insertions and deletions were distinct from the SNP dataset, and contained many genes involved in pathways related to both abiotic and biotic stress responses, which has been observed before in diverse bioenergy sorghums (Songsomboon et al., 2021). Additionally our study identified structural variants affecting genes involved in tRNA nucleoside modifications, programmed cell death in response to symbionts, and photosynthetic light response, all of which were previously identified by other studies as GO terms of interest in relation to sorghum stress response (Ortiz et al., 2017; Wang et al., 2017).

SVs strictly occurring in either sweet or non-sweet genotypes also offer unique insights into the differences between these types that could be key to dissecting differences in carbon allocation in sorghum. Of particular interest is the fact that SVs restricted to sweet sorghum genotypes affected many genes related to metal metabolism and iron transport. This connection between iron transport and sugar accumulation has been observed in other comparative genomic studies of sorghum (2020; Brenton et al., 2016; Cooper et al., 2019), and appears to be a key factor distinguishing sweet sorghums from both cellulosic and grain types.

Over a third of protein coding genes and over 75 percent of noncoding genes annotated in this study did not map back to the Btx623 reference genome. With a growing number of studies illustrating the importance of noncoding DNA and RNA as potential regulatory elements (Waititu et al., 2020), it is evident that large pan-genome annotations are vital in quickly identifying and annotating potential regulatory ‘pseudo-genes’ as well as protein coding genes that are divergent from the common reference. Previous pan-genome studies in sorghum and maize have identified high levels of gene content variation, with 53-64 percent of genes identified as non-core (Tao et al., 2021; Ruperao et al., 2021; Hufford et al., 2021). We corroborate these findings with about 63 percent of our genes being identified as either shell or cloud to our population, despite this particular population lacking wild representation, indicating relatively high amounts of latent variation, even among domesticated varieties of sorghum.

Taken together, our results demonstrate the value of exploring genome-wide patterns of both SNPs and larger structural variants to gain new insights into the genetic architectures of complex and agronomically important traits. To advance both sorghum breeding efforts and our understanding of crop plant evolution, we have generated this new extensive dataset that is publicly available through SorghumBase (Gladman et al., 2022) and which can be readily integrated into an already valuable genetic resource for future mapping studies.

## Data availability statement

The datasets presented in this study can be found in online repositories. The names of the repository/repositories and accession number(s) can be found below: https://www.ebi.ac.uk/ena, PRJEB55613 https://ftp.sorghumbase.org/Voelker_et_al_2022/, N/A.

## Author contributions

WV: Writing, variant analysis, created figures and tables, performed scaffolding. KK: Performed TE analysis, alignments, variant calling and wrote corresponding methods sections. LA: Aided in scripting of figure creation and filtering of variants. KS: Growing and DNA Extraction of plant material. CP: Aided in genome assembly. KC, ZL, AO: Gene and transposable element annotations. DW: Experimental design, writing. ZB: designed CP-NAM population, provided genetic materials JB: development and release of CP-NAMs. EC: Writing, created figures, conceived the project, advised, and helped direct analysis. All authors contributed to the article and approved the submitted version.
