# Supplementary material for: Ten new high-quality genome assemblies for diverse bioenergy sorghum genotypes
Source: Front Plant Sci. 2023 Jan 4;13:1040909. doi: 10.3389/fpls.2022.1040909 (PMC9846640; doi:10.3389/fpls.2022.1040909)
Supplement: Supplementary file 1 [file DataSheet_1.docx]

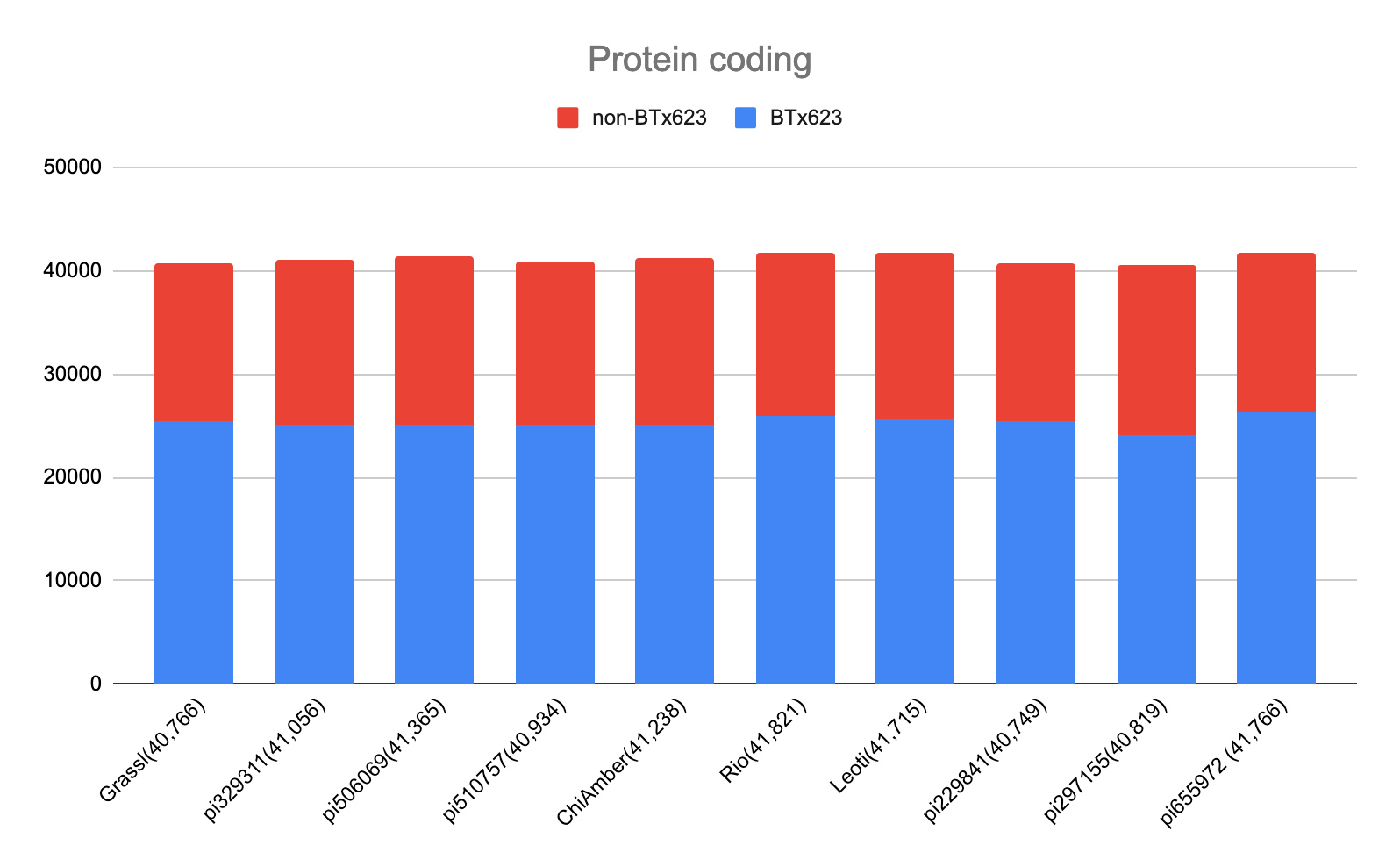


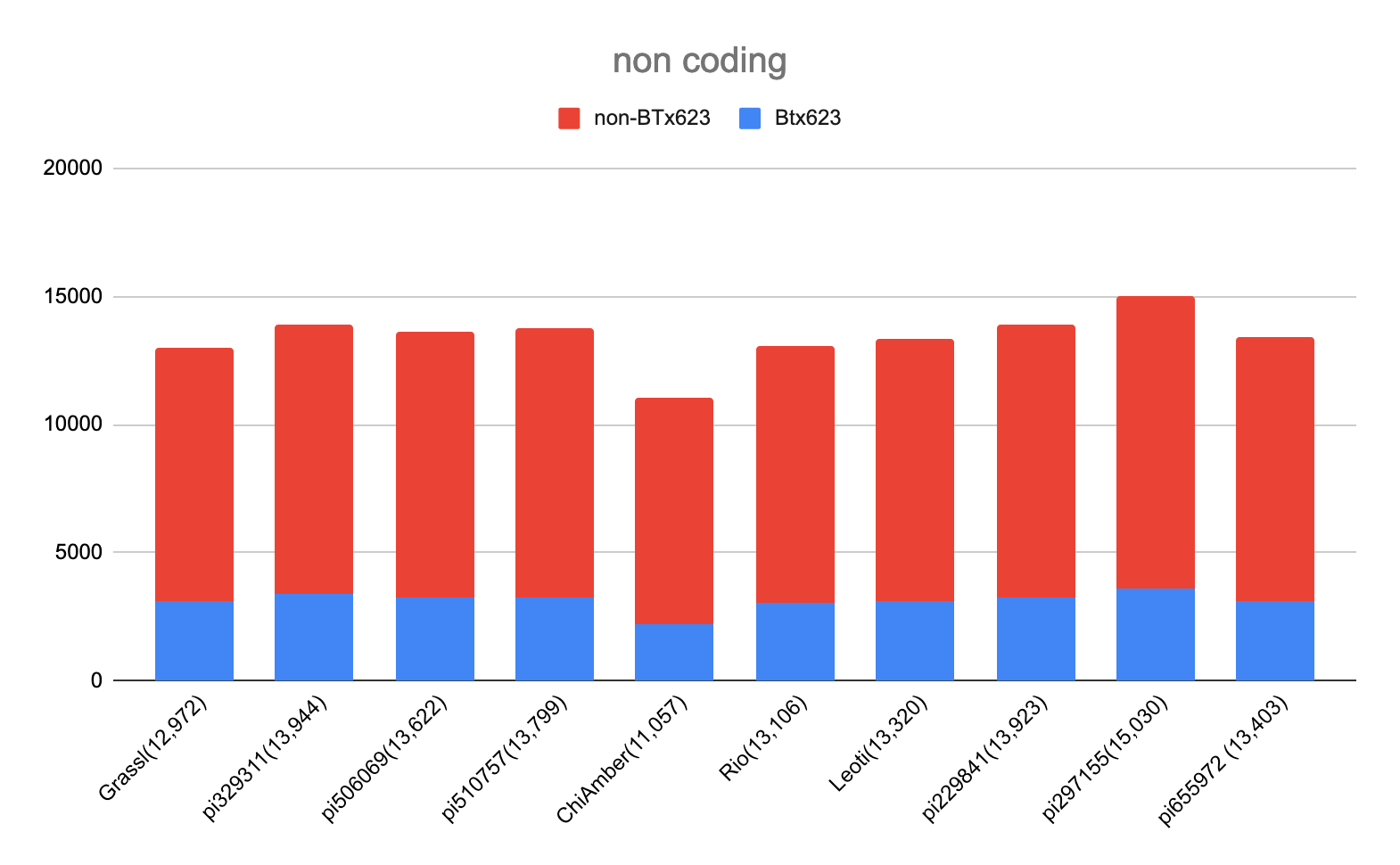


Supplemental Figure 1a. Distribution of Btx623 genes and non-BTX623 genes across the protein and non coding Sorghum annotations


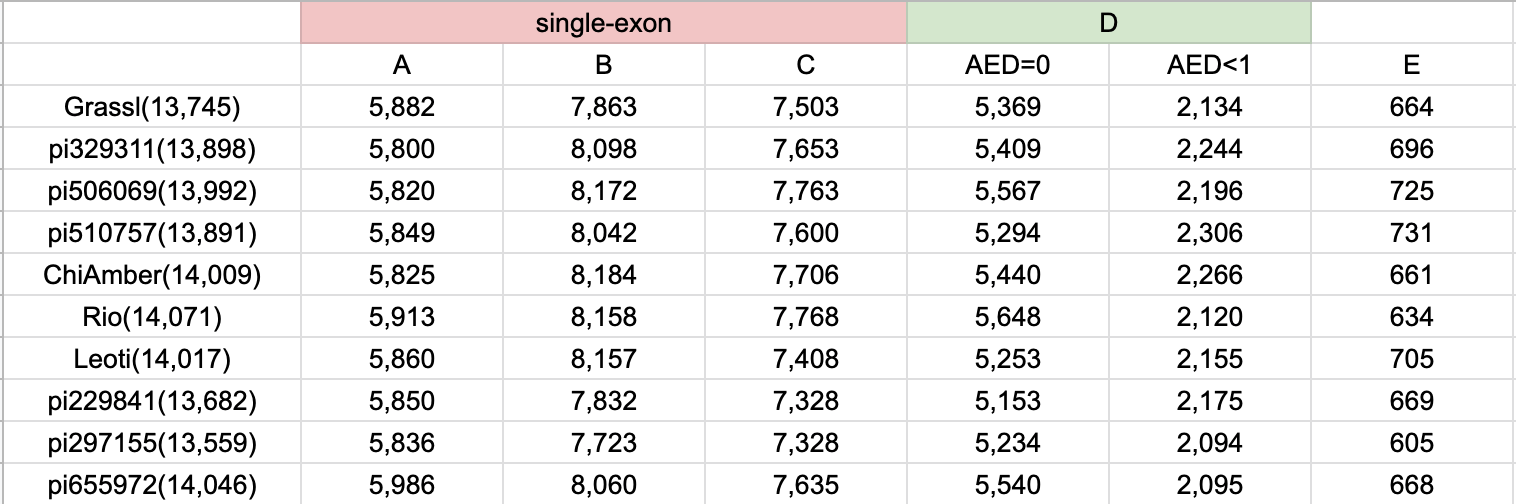
Supplemental Figure 1b.  Single exon gene and Annotation Edit Distance. Column A shows counts of single exon genes in BTx623. Column B shows counts of single exon genes that are not found in Btx623, column C represents the number of single exon genes that are annotated in other sorghum lines whether these are in Btx623 gene trees, or are found in sorghum lineage specific gene trees.
