# Supplementary material for: Ten new high-quality genome assemblies for diverse bioenergy sorghum genotypes
Source: Front Plant Sci. 2023 Jan 4;13:1040909. doi: 10.3389/fpls.2022.1040909 (PMC9846640; doi:10.3389/fpls.2022.1040909)
Supplement: Supplementary file 2 [file DataSheet_2.docx]

**A.**
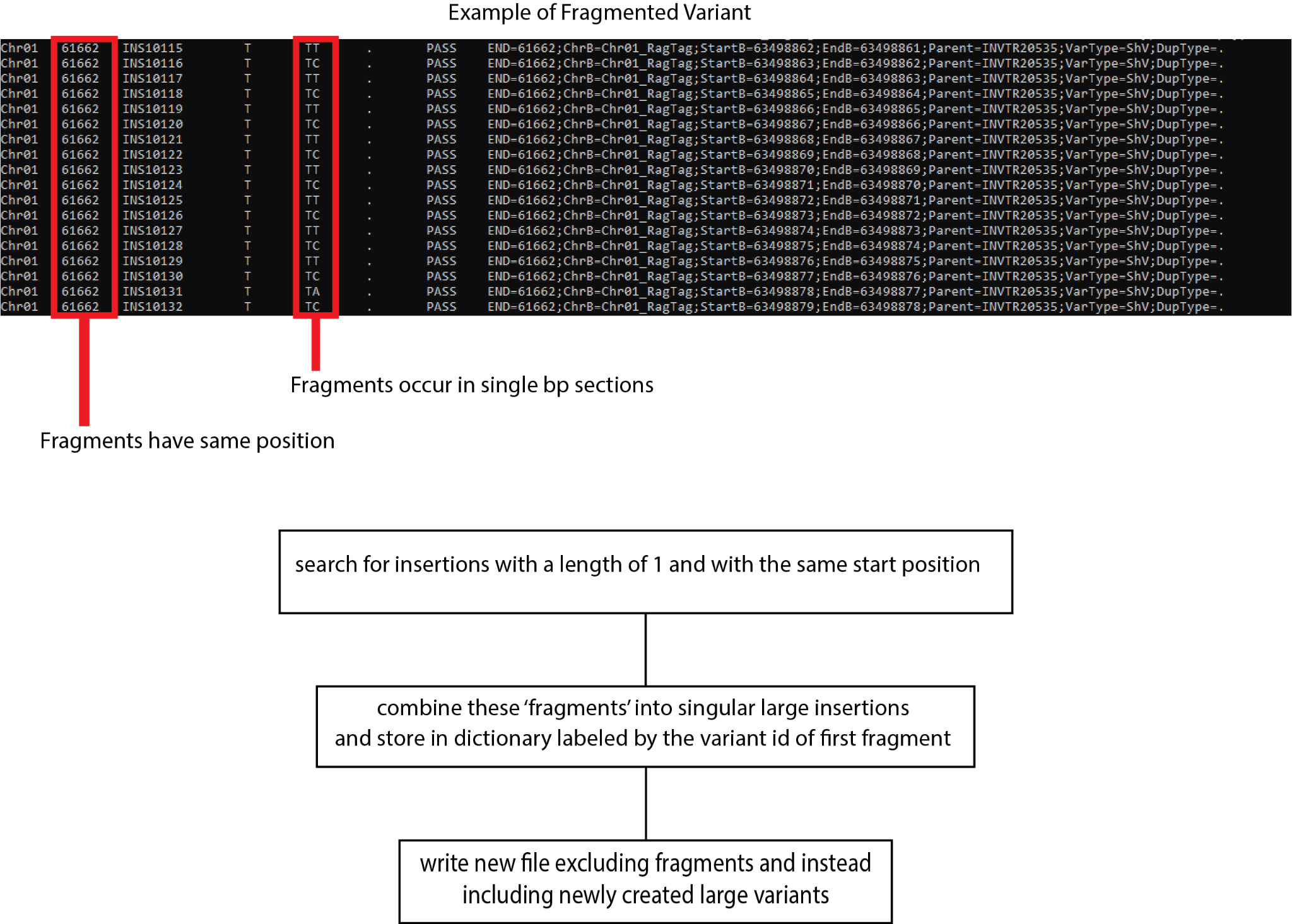


**B.**

**Step 1.**

**Step 2.**

**Step 3.**

Supplementary Figure 2. **A.** A screenshot of an insertion in the VCF output created by Syri that has been incorrectly fragmented into single nucleotide pieces. Red boxes indicate identical start positions and variant length respectively, which are both used to easily identify fragmented insertions called by syri. **B.** Our pipeline used to combine fragmented variants into single cohesive variants. First fragments are identified using custom scripts following the rules of Step 1. In Step 2 the fragments are combined into one variant and the information is stored. Step 3 is the writing of the new Insertion file including all unmodified insertions as well as newly created large variants, excluding the fragments used to create them.
