# Supplementary material for: Ten new high-quality genome assemblies for diverse bioenergy sorghum genotypes
Source: Front Plant Sci. 2023 Jan 4;13:1040909. doi: 10.3389/fpls.2022.1040909 (PMC9846640; doi:10.3389/fpls.2022.1040909)
Supplement: Supplementary file 3 [file DataSheet_3.docx]

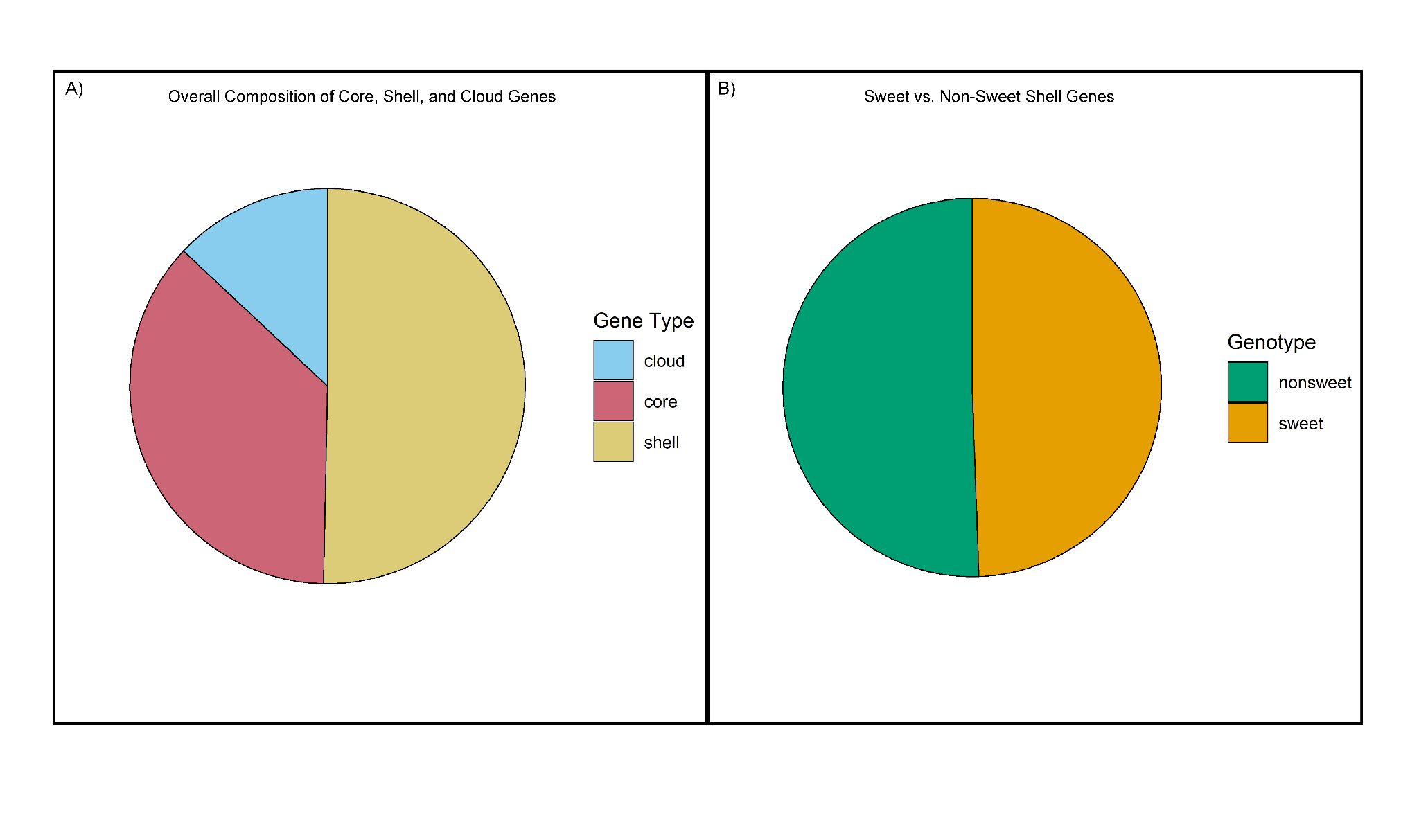


Supplementary Figure 3a: Gene Presence/Absence and Composition. The composition of genes that are cloud (in blue), shell (in yellow) and core (in red). Core genes are present in all 10 genotypes, shell genes are present in 2-9 genotypes, and cloud genes are only present in 1 genotype.

Supplementary Figure 3
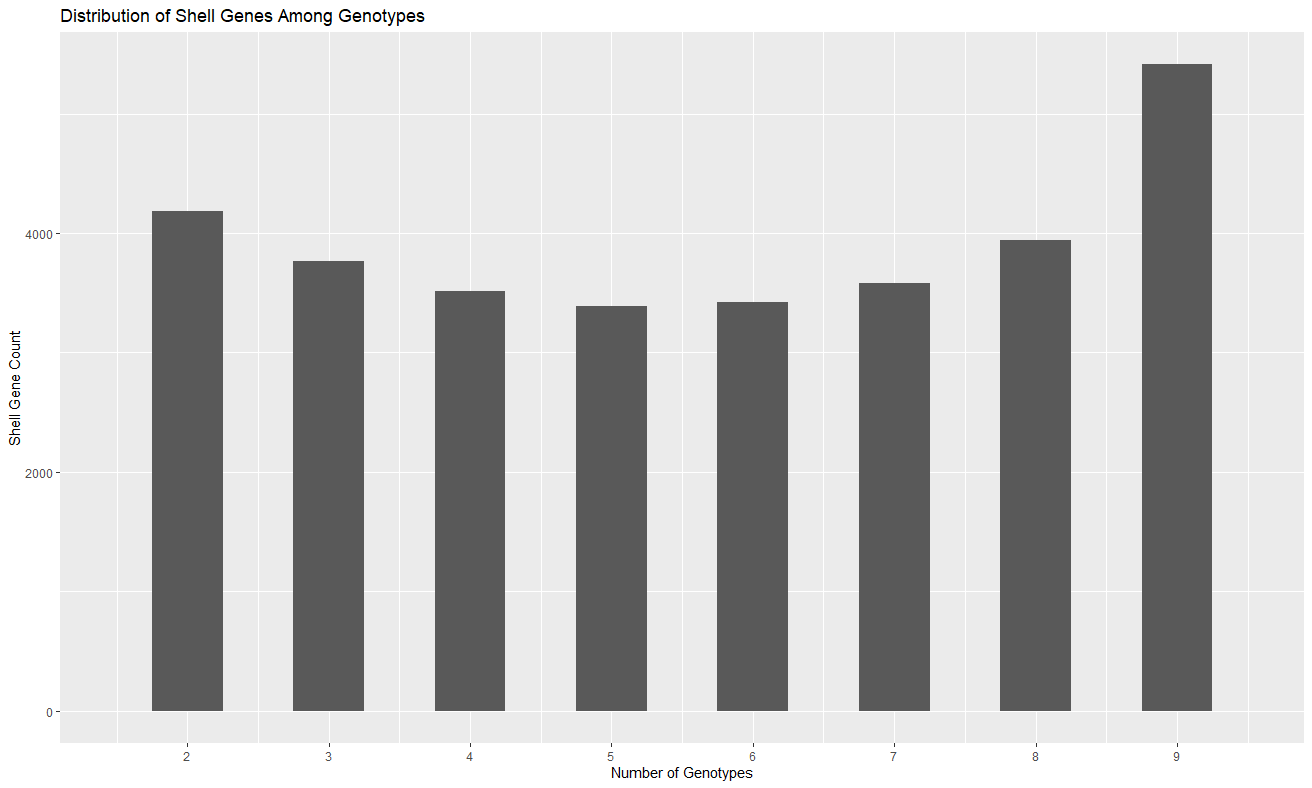
b: Distribution of Shell Genes Among Genotypes. A bar plot visualizing the distribution of shell genes among genotypes. The y-axis describes the number of shell genes present, where the x-axis describes the number of genotypes they are present in.
