# Supplementary material for: Ten new high-quality genome assemblies for diverse bioenergy sorghum genotypes
Source: Front Plant Sci. 2023 Jan 4;13:1040909. doi: 10.3389/fpls.2022.1040909 (PMC9846640; doi:10.3389/fpls.2022.1040909)
Supplement: Supplementary file 4 [file DataSheet_4.docx]

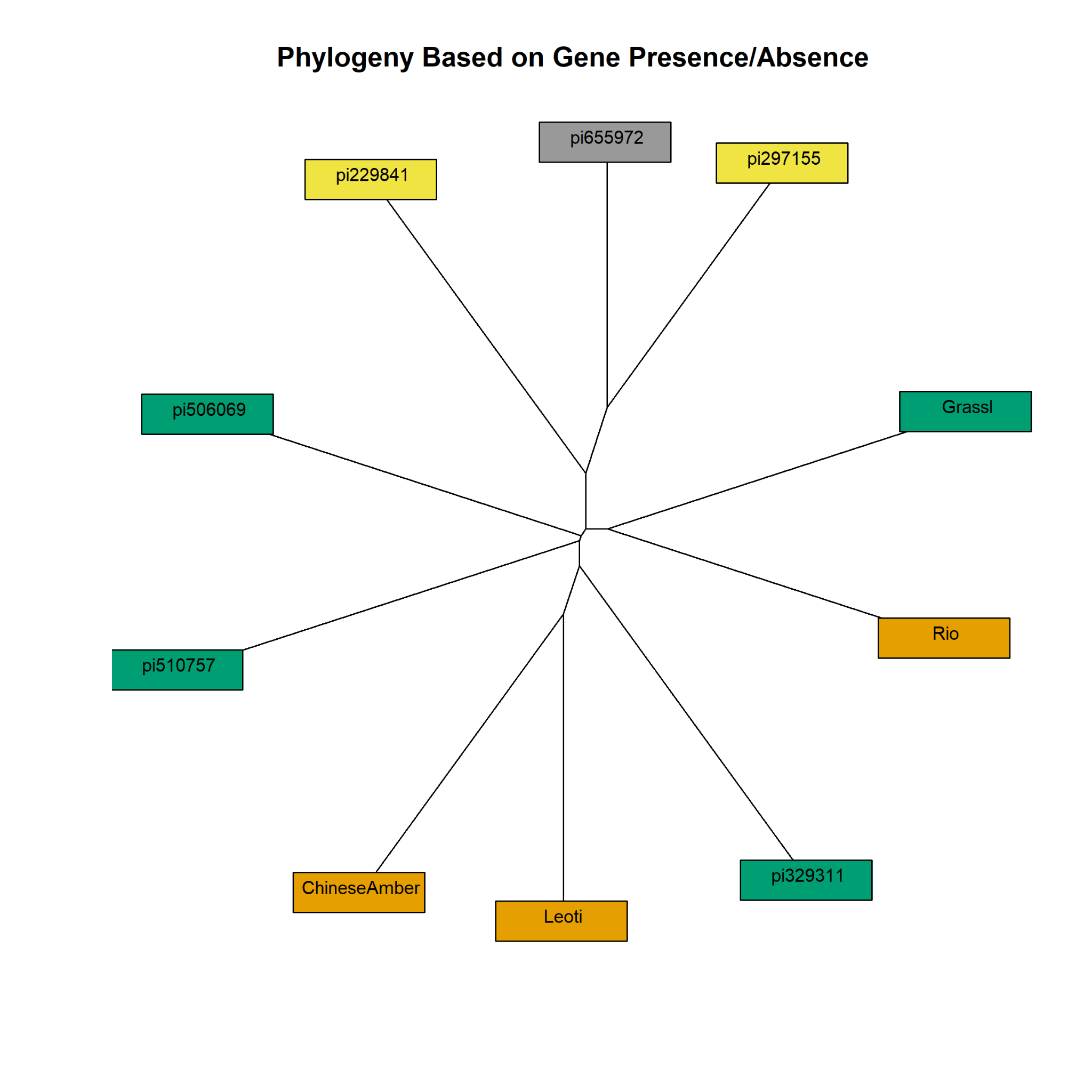


Supplementary Figure 4a: Distance tree calculated from gene presence/absence. Ends that are in orange are sweet-type, those in yellow are grain-type, those in gray are forage-type and those in green are cellulosic-type.


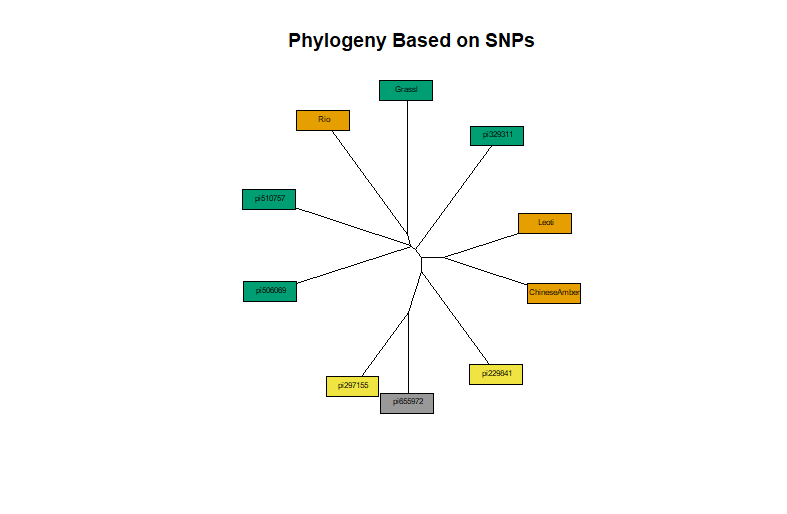


Supplementary Figure 4b: Distance tree calculated from the presence/absence of SNPs called by the program Syri. The ends that are in orange are sweet-type, those in yellow are grain-type, those in gray are forage-type, those in green are cellulosic-type.
