# Supplementary material for: Ten new high-quality genome assemblies for diverse bioenergy sorghum genotypes
Source: Front Plant Sci. 2023 Jan 4;13:1040909. doi: 10.3389/fpls.2022.1040909 (PMC9846640; doi:10.3389/fpls.2022.1040909)
Supplement: Supplementary file 5 [file DataSheet_5.docx]

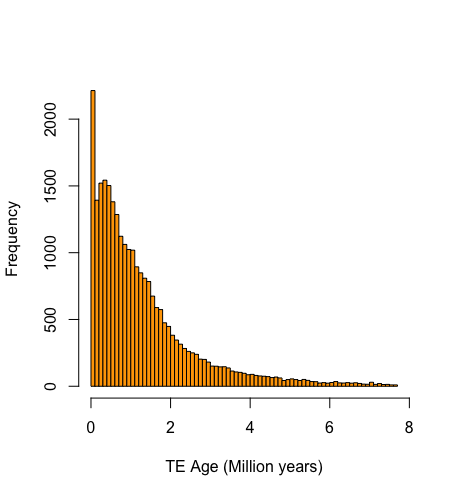

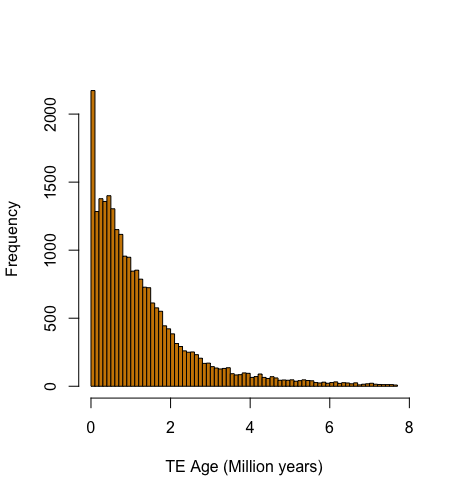

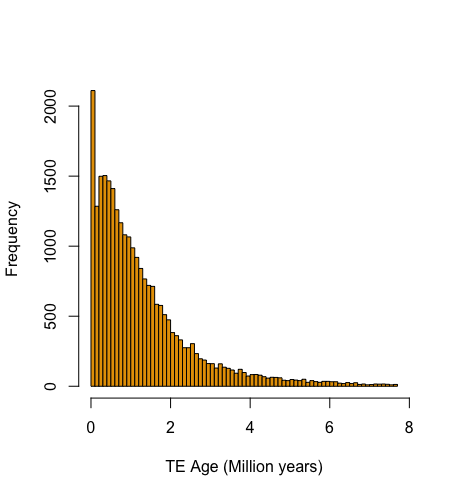


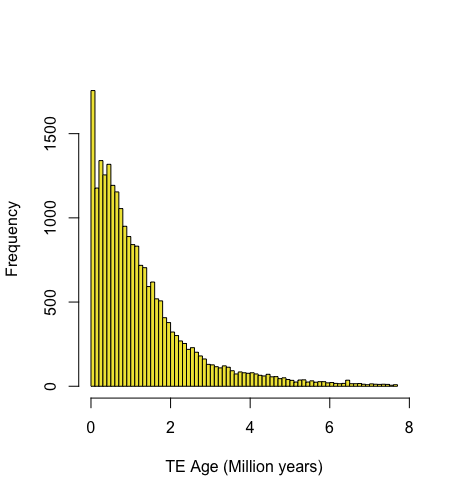

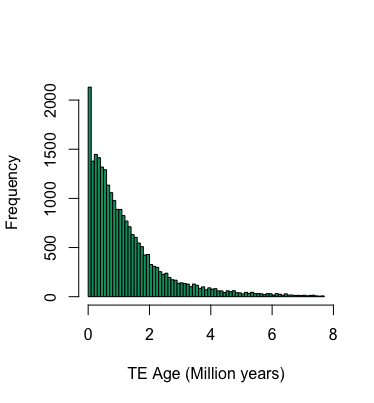

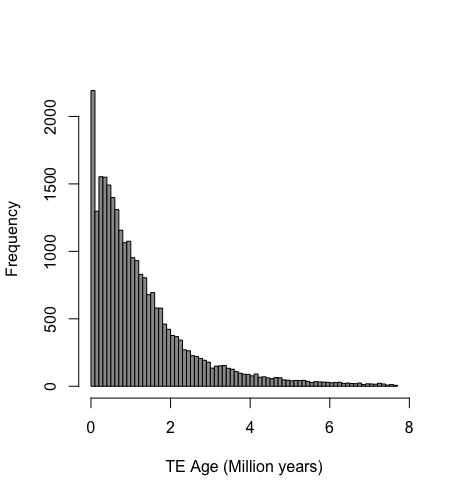

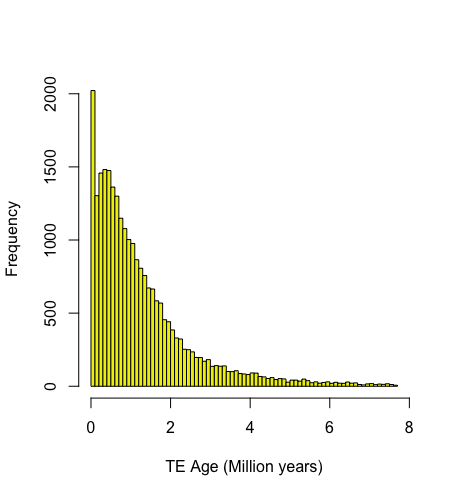

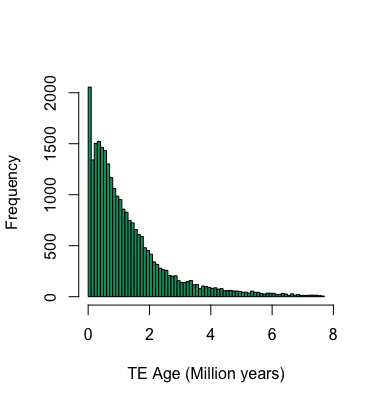

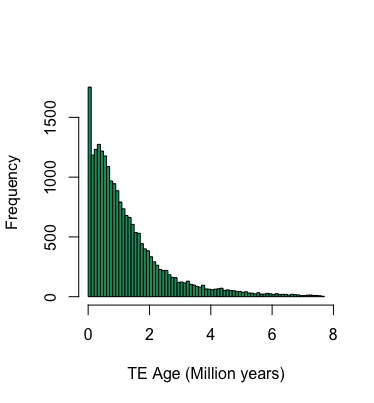

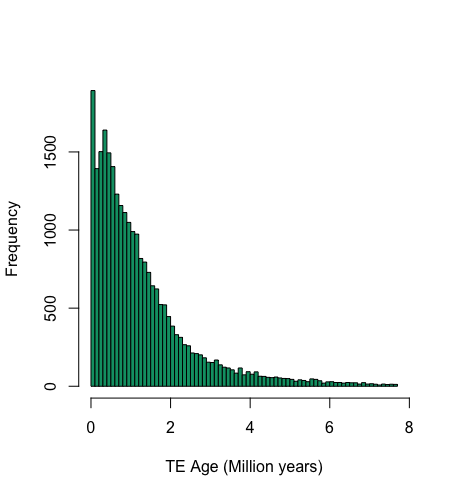


Supplemental figure 5: Frequency distribution plots showing the distribution of TE ages in each individual sorghum genotype. TE age is shown in millions of years. Colors represent the types of sorghums-cellulosic (green), forage (gray), sweet (orange) and grain (yellow).
