# Supplementary material for: Ten new high-quality genome assemblies for diverse bioenergy sorghum genotypes
Source: Front Plant Sci. 2023 Jan 4;13:1040909. doi: 10.3389/fpls.2022.1040909 (PMC9846640; doi:10.3389/fpls.2022.1040909)
Supplement: Supplementary file 6 [file DataSheet_6.docx]

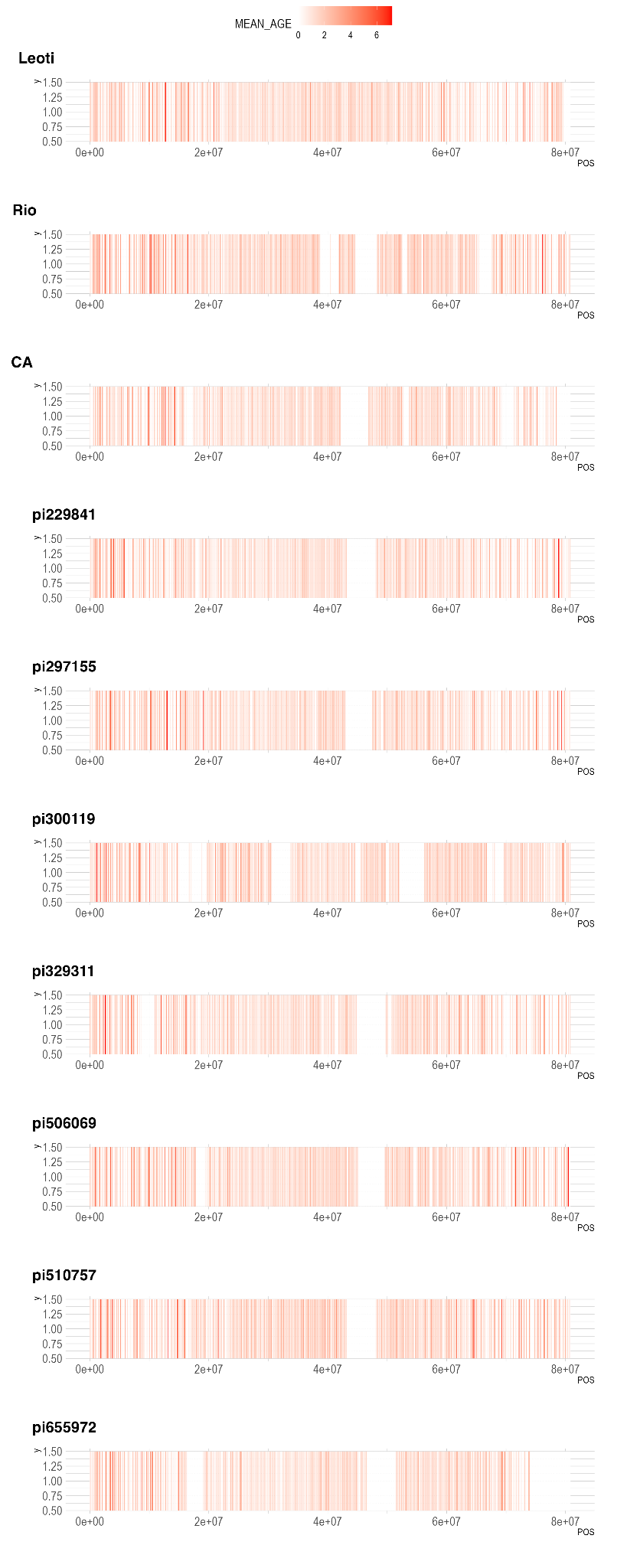


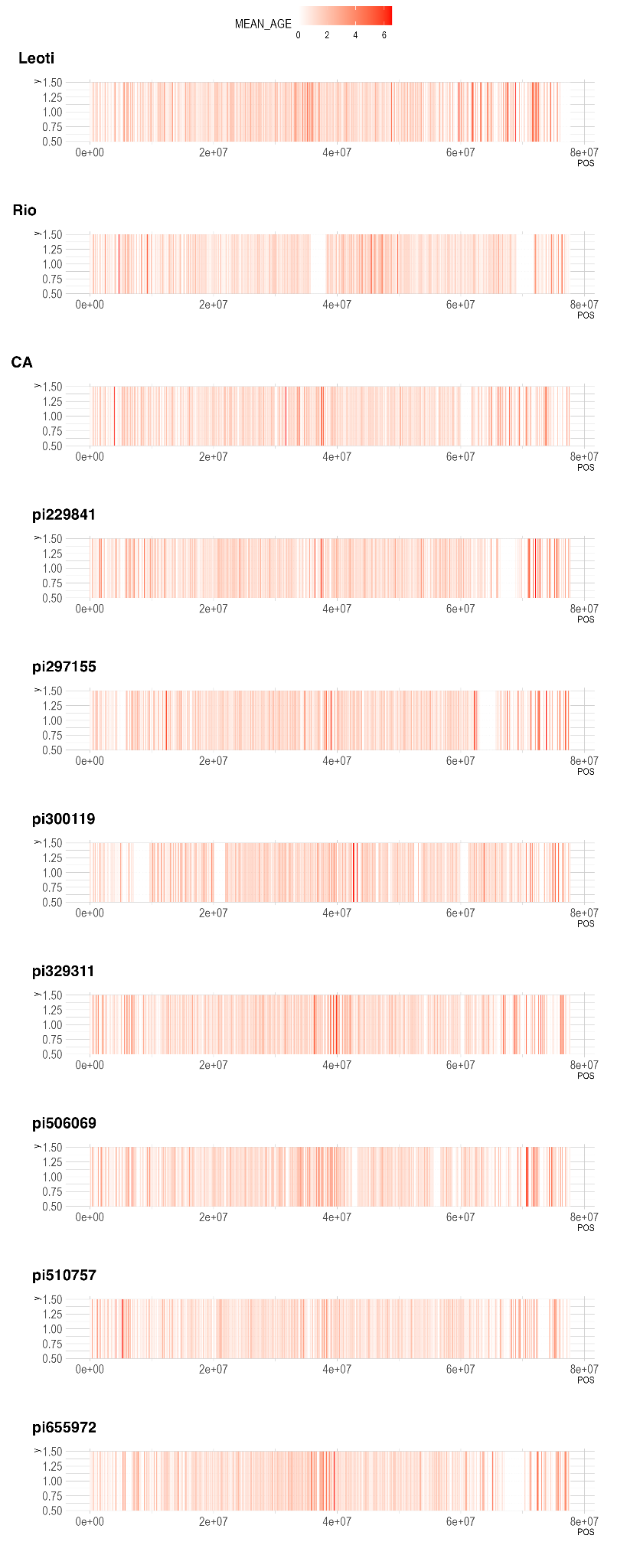


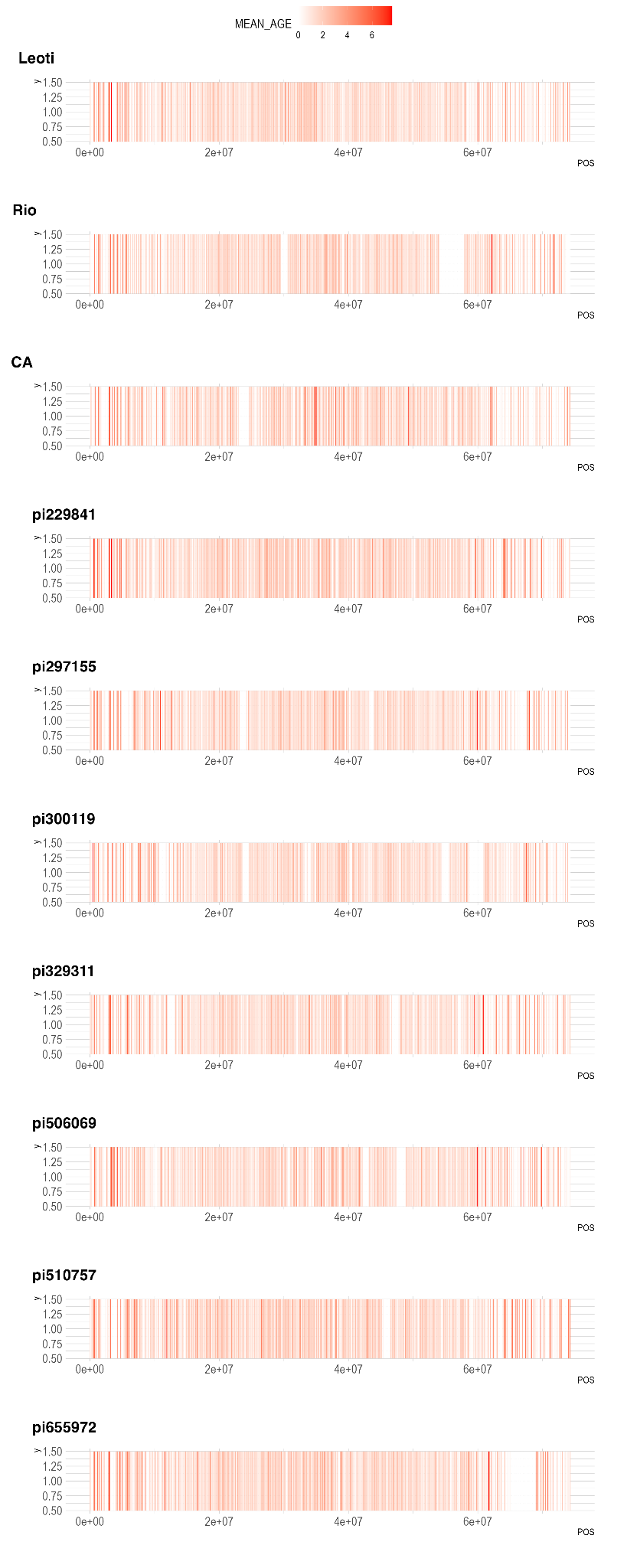


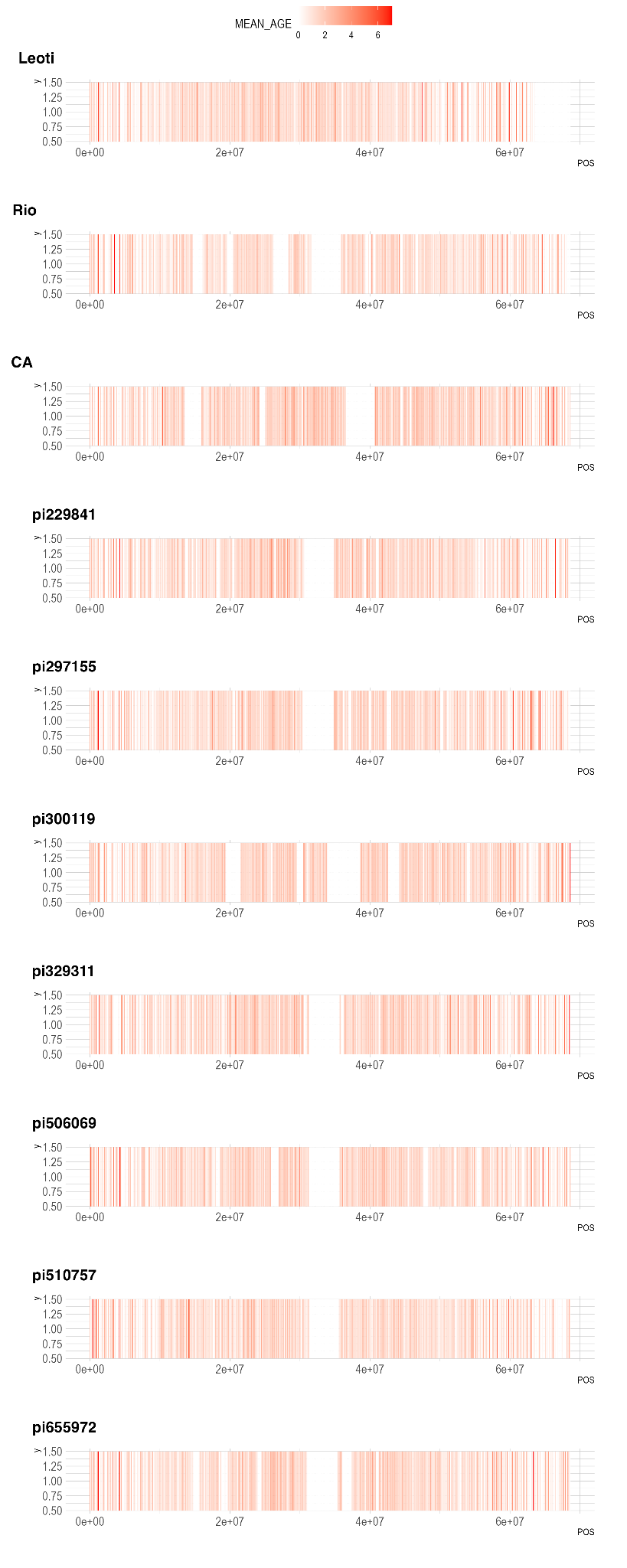


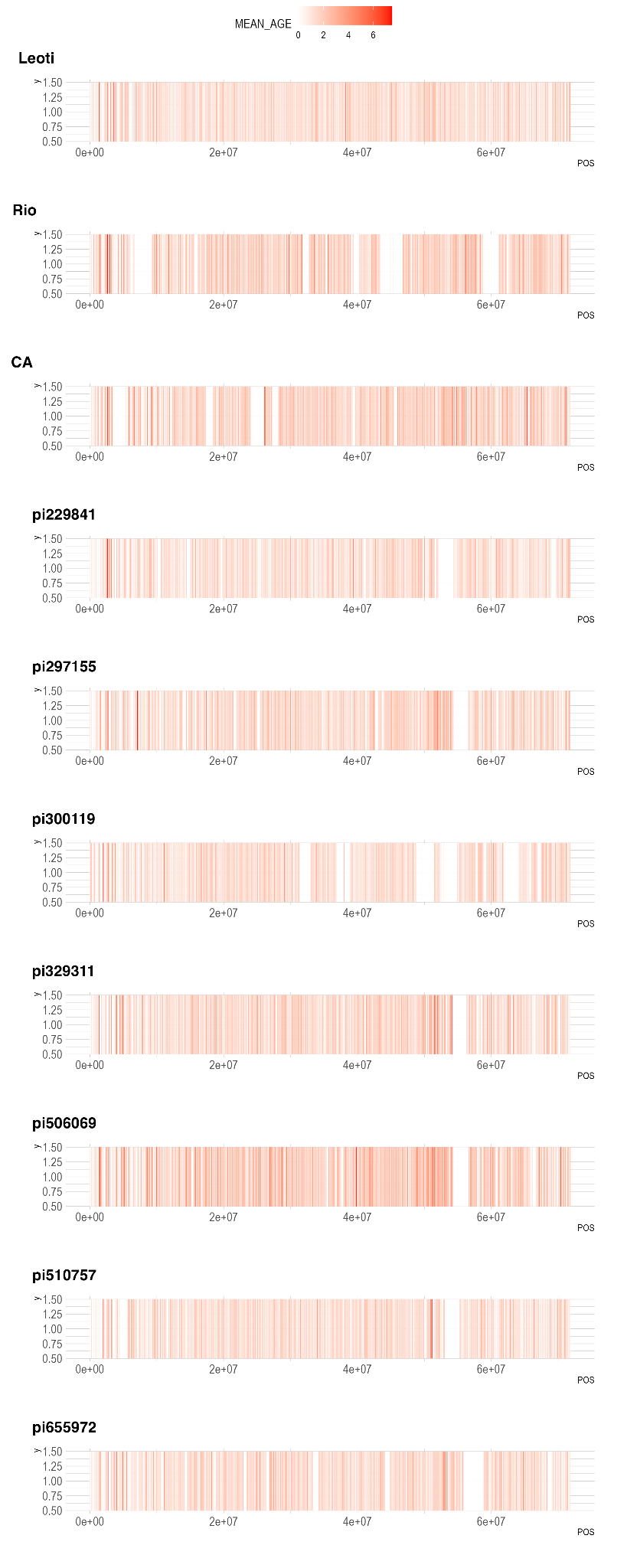


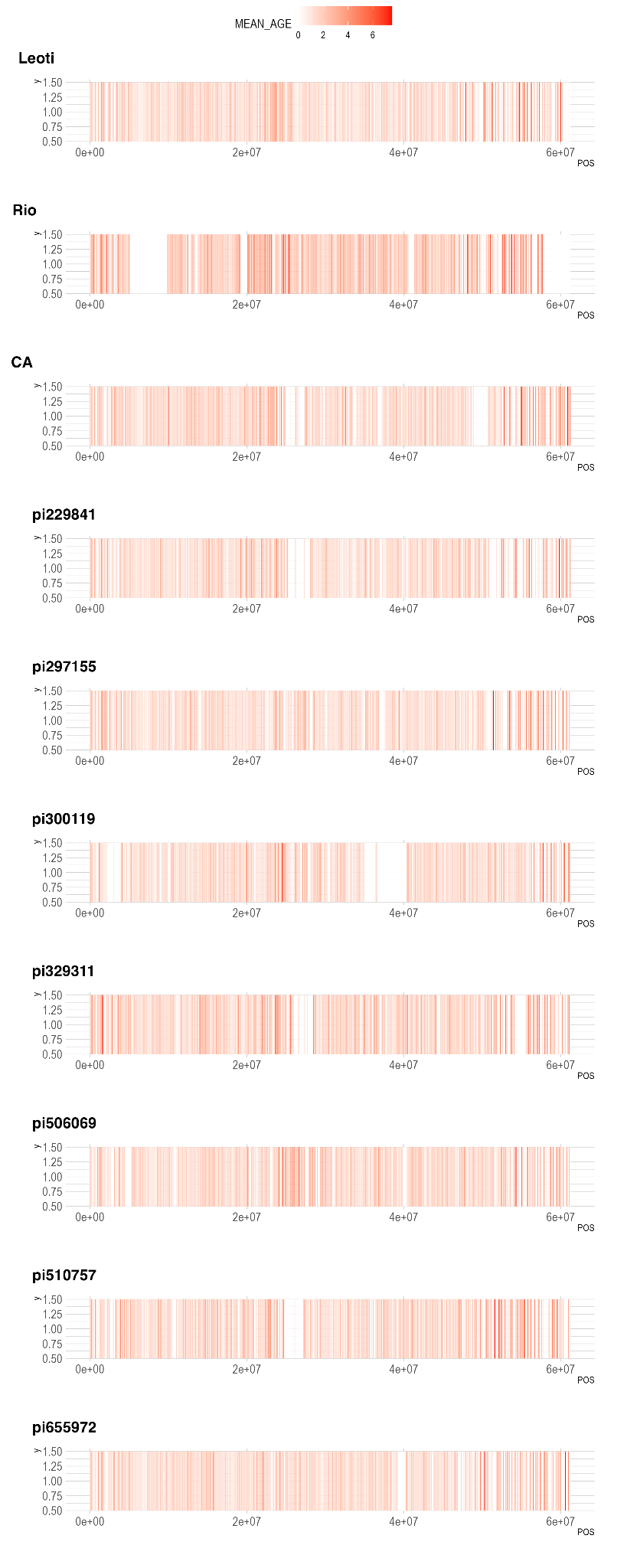


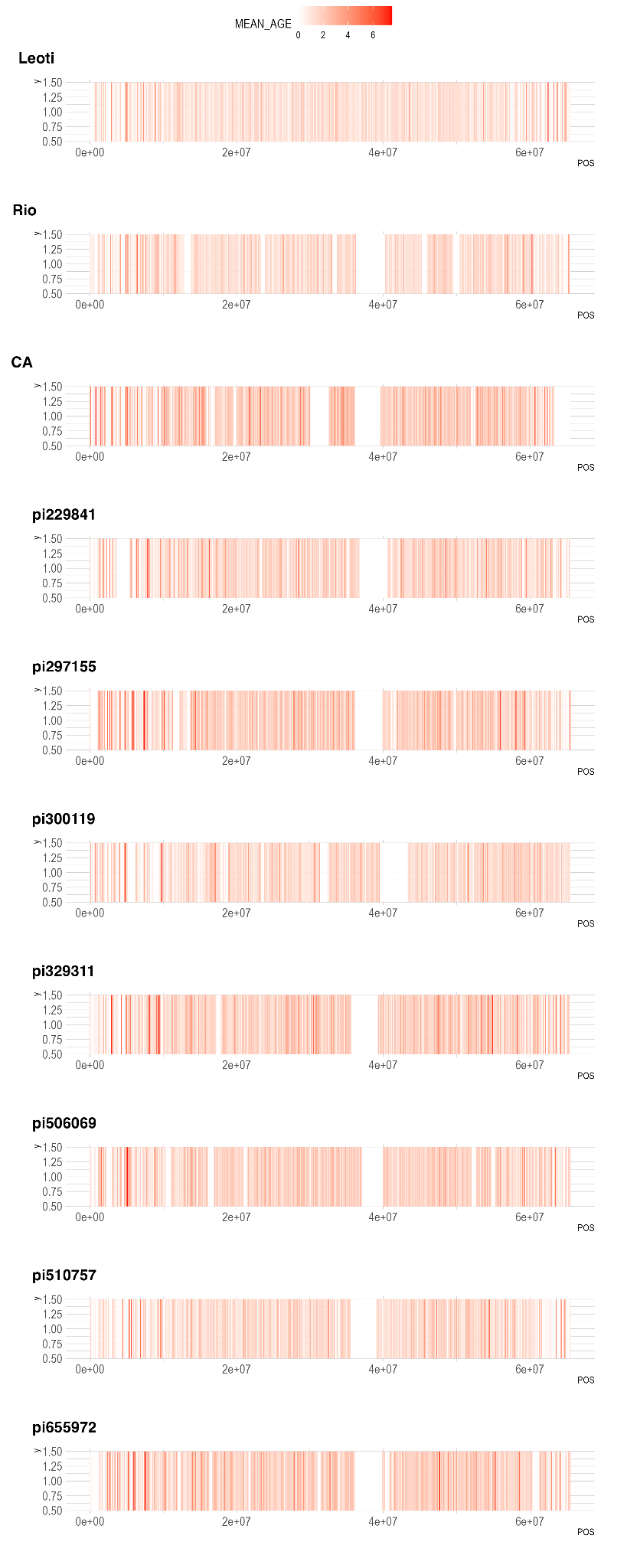


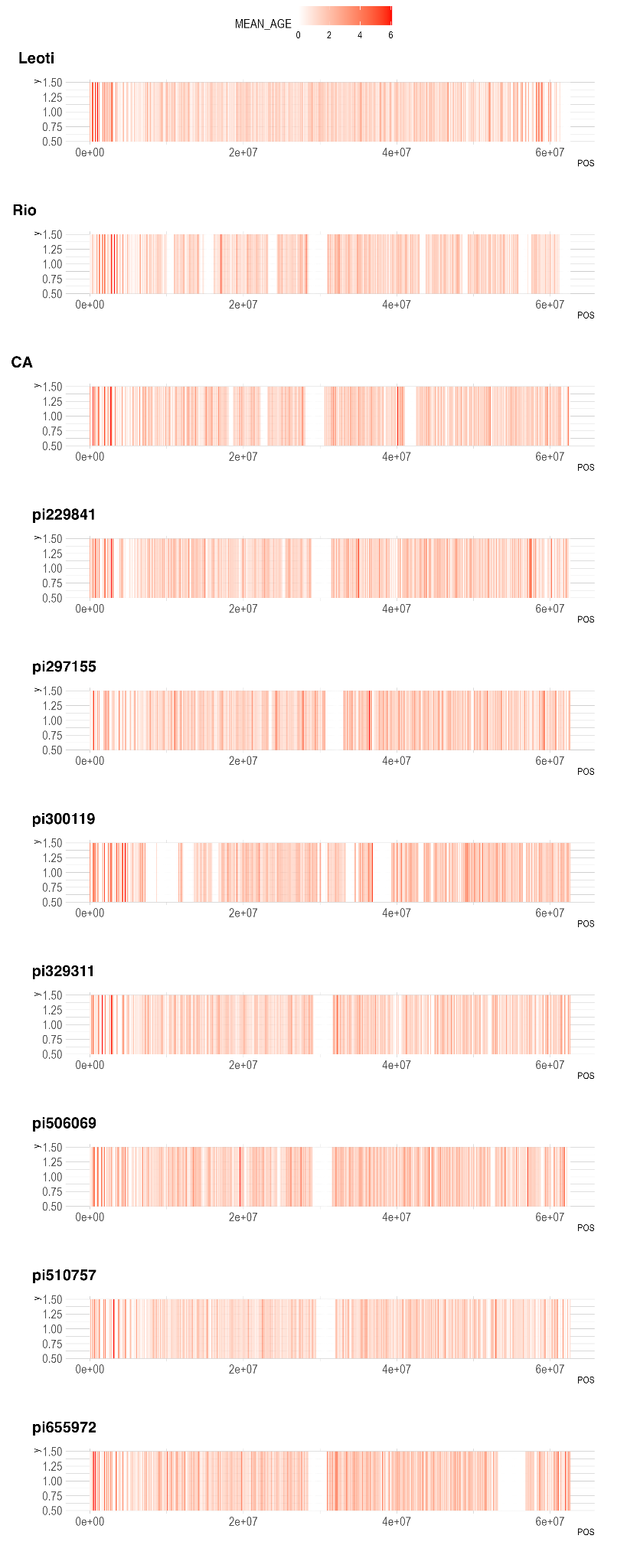


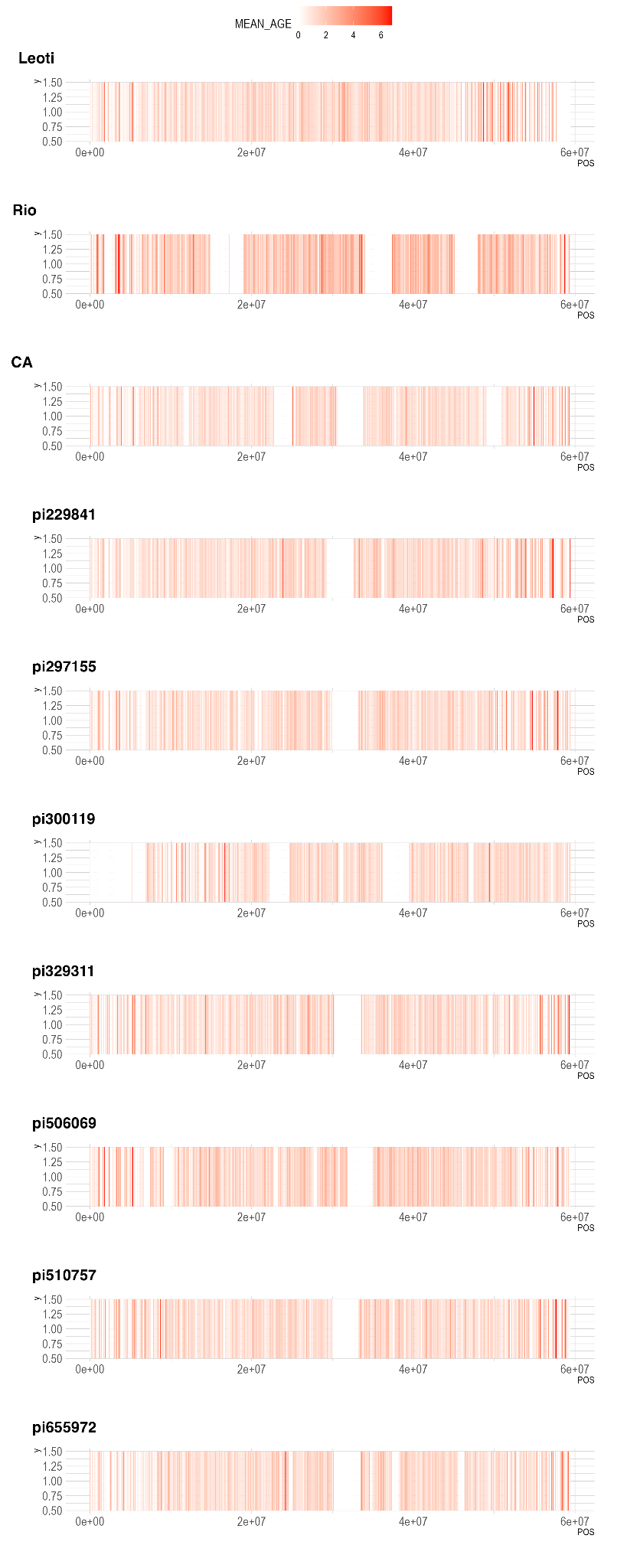


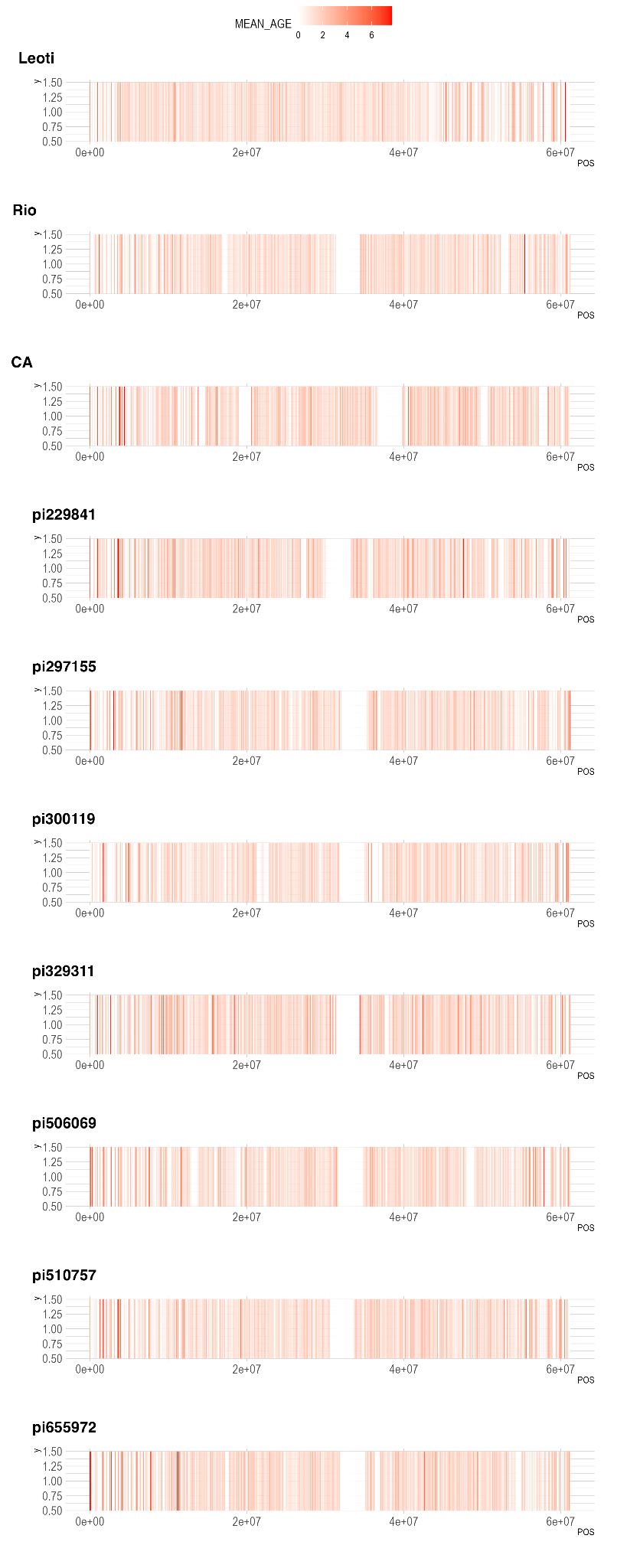


Supplemental figure 6: Heat maps showing the age of TEs averaged in 100kb non-overlapping sliding windows across all the genotypes. Figures A-J represent chromosomes 1-10 respectively for all the genomes. The X-axis represents the chromosome positions (POS).
