# Supplementary material for: Ten new high-quality genome assemblies for diverse bioenergy sorghum genotypes
Source: Front Plant Sci. 2023 Jan 4;13:1040909. doi: 10.3389/fpls.2022.1040909 (PMC9846640; doi:10.3389/fpls.2022.1040909)
Supplement: Supplementary file 8 [file Table_2.docx]

Supplemental Table 2. Assembly Statistics for NAM Parent Genotypes

| Name | Raw Read Count | Expected Coverage | Contig N50 | Contig L90 | Scaffolded Genome Size (bp) | Genes in Scaffolds |
| --- | --- | --- | --- | --- | --- | --- |
| Grassl | 2,375,877 | 31x | 236,204 | 2940 | 641,452,936 | 30,009 |
| Chinese Amber | 2,220,606 | 35x | 2,281,751 | 355 | 698,625,432 | 29,078 |
| PI229841 | 1,819,255 | 28x | 433,895 | 1811 | 669,663,635 | 29,742 |
| PI297155 | 2,326,510 | 31x | 720,856 | 1118 | 679,888,921 | 30,440 |
| PI329311 | 2,137,532 | 30x | 302,551 | 2803 | 686,114,297 | 28,908 |
| PI506069 | 2,149,205 | 32x | 1,403,415 | 662 | 699,327,342 | 29,028 |
| PI510757 | 1,856,867 | 24x | 176,247 | 4129 | 670,133,795 | 29,115 |
| Rio | 2,443,144 | 38x | 3,167,246 | 277 | 709,117,272 | 30,249 |
| Leoti | 2,470,190 | 40x | 3,012,307 | 284 | 626,432,001 | 29,578 |
| PI655972 | 2,903,110 | 42x | 1,771,065 | 511 | 696,083,882 | 30,666 |

Expected coverage was calculated based on read counts, read length, and the BTx623 reference genome size (708Mbp).
